# Enzymatic fermentation of rapeseed cake significantly improved the soil environment of tea rhizosphere

**DOI:** 10.1186/s12866-023-02995-7

**Published:** 2023-09-07

**Authors:** Yujie Song, Litao Sun, Huan Wang, Shuning Zhang, Kai Fan, Yilin Mao, Jie Zhang, Xiao Han, Hao Chen, Yang Xu, Kangwei Sun, Zhaotang Ding, Yu Wang

**Affiliations:** 1 Tea Research Institute, Qingdao Agricultural University, Qingdao, 266109 China; 2 Tea Research Institute, Shandong Academy of Agricultural Sciences, Jinan, 250100 China

**Keywords:** *Camellia sinensis*, Rapeseed cake, Rhizosphere microorganisms, Soil metabolites

## Abstract

**Background:**

Rapeseed cake is an important agricultural waste. After enzymatic fermentation, rapeseed cake not only has specific microbial diversity but also contains a lot of fatty acids, organic acids, amino acids and their derivatives, which has potential value as a high-quality organic fertilizer. However, the effects of fermented rapeseed cake on tea rhizosphere microorganisms and soil metabolites have not been reported. In this study, we aimed to elucidate the effect of enzymatic rapeseed cake fertilizer on the soil of tea tree, and to reveal the correlation between rhizosphere soil microorganisms and nutrients/metabolites.

**Results:**

The results showed that: (1) The application of enzymatic rapeseed cake increased the contents of soil organic matter (OM), total nitrogen (TN), total phosphorus (TP), available nitrogen (AN), and available phosphorus (AP); increased the activities of soil urease (S-UE), soil catalase (S-CAT), soil acid phosphatase (S-ACP) and soil sucrase (S-SC); (2) The application of enzymatic rapeseed cake increased the relative abundance of beneficial rhizosphere microorganisms such as *Chaetomium*, *Inocybe*, *Pseudoxanthomonas*, *Pseudomonas*, *Sphingomonas*, and *Stenotrophomonas*; (3) The application of enzymatic rapeseed cake increased the contents of sugar, organic acid, and fatty acid in soil, and the key metabolic pathways were concentrated in sugar and fatty acid metabolisms; (4) The application of enzymatic rapeseed cake promoted the metabolism of sugar, organic acid, and fatty acid in soil by key rhizosphere microorganisms; enzymes and microorganisms jointly regulated the metabolic pathways of sugar and fatty acids in soil.

**Conclusions:**

Enzymatic rapeseed cake fertilizer improved the nutrient status and microbial structure of tea rhizosphere soil, which was beneficial for enhancing soil productivity in tea plantations. These findings provide new insights into the use of enzymatic rapeseed cake as an efficient organic fertilizer and expand its potential for application in tea plantations.

**Supplementary Information:**

The online version contains supplementary material available at 10.1186/s12866-023-02995-7.

## Introduction

Tea plant (*Camellia sinensis*) is an important cash crop for leaves, which is widely cultivated in tropical and subtropical regions. Fertilization is the most direct and effective way to improve the yield and quality of tea, and different fertilizer types and amounts of fertilizers have a significant impact on it [1–4]. The application of organic fertilizer is one of the important measures to improve the soil environment of tea plantations, enhance soil productivity, and improve tea yield and quality [5–9]. However, due to the slow effectiveness of organic fertilizers, it is difficult to meet the nutrient demand of tea tree growth on time. And at the same time, to improve the yield and quality, the application of organic fertilizers is often increased, thus leading to the high cost of organic fertilizer application. Therefore, from the perspective of nutrient effectiveness and economy, the raw materials of organic fertilizer are applied after fermentation, which not only improves the structure and function of soil microorganisms in a short time but also achieves the effect of quick-acting fertilizer. This is beneficial for improving the yield and quality of tea. Previous studies in our laboratory have shown that fermented soybeans as fertilizers can improve soil nutrient status, regulate related microbial communities, and positively affect the budding of tea [10]. This study laid the foundation for our further application of enzymatically fermented organic materials in tea plantations.

Rapeseed cake is an important agricultural waste containing a large amount of nutrients and is one of the commonly used organic fertilizers in tea gardens. Previous studies have shown that applying rapeseed cake fertilizer in tea gardens could reduce the emissions of greenhouse gas, promote the sequestration of soil carbon, and increase the yields of tea [11–14]. Mixed application of rapeseed cake fertilizer and green manure could maintain soil organic nutrients (total organic carbon, total phosphorus, and available phosphorus) and increase soil nutrients and bacterial diversity [15, 16]. Mixed application of rapeseed cake and mineral fertilizers could improve the soil's physical environment, enhance root proliferation and increase the biomass of tea [17].

However, in practice, rapeseed cake as a fertilizer is mainly applied directly, which is difficult to exert its optimal fertilizer effect. The effects of metabolites, microbial community structure, and their interrelationships in the rhizosphere soil after the application of rapeseed cake fertilizer in tea plantations have not been reported.

At present, our research team has studied the physical and chemical properties, microbial community structure, and metabolites of rapeseed cake after natural fermentation and enzymatic fermentation, and has made important progress [18]. The research showed that the microbial diversity of rapeseed cake fermented by enzyme was significantly increased and had specificity. The relative abundance of *Bacillus*, *Lysinibacillus*, *Empedoactor*, etc. was significantly higher than that of natural fermentation. Enzymatic fermentation promoted the transformation of macromolecular substances in rapeseed cake, and increased small molecule metabolites. The enrichment pathways of metabolites mainly focused on sugar metabolism and fatty acid metabolism. This study laid a good foundation for further development of using rapeseed cake as organic fertilizer.

In order to further explore the effects of enzymatic rapeseed cake on the microorganisms and metabolites in the rhizosphere soil of tea plants, we studied the physical and chemical properties of the soil, evaluated the community structure of the microorganisms in the rhizosphere soil, revealed the composition of soil metabolites and their regulatory pathways, and provided a theoretical basis for the future application of enzymatic rapeseed cake as organic fertilizer in tea gardens.

## Results and analysis

### Effects of different fertilization treatments on microorganisms of rhizosphere soil

#### Effect of different fertilization treatments on the total number of bacteria and fungi

In order to quantify the biomass of soil microorganisms under different fertilization treatments, we cultured bacteria and fungi of each treatment, and counted colony-forming units (CFUs), respectively. The results showed that the total number of bacteria in REF was significantly higher than the other three treatment (*p* < 0.05), while the total number of fungi in RNF was significantly lower than the other three treatment groups (*p* < 0.05) (Table 1). As expected, the application of enzymatic rapeseed cake increased the number of microbial communities (especially bacterial communities) in the soil, altering the structure of microbial communities.

**Table 1:** Total number of bacteria and fungi under different fertilization treatments

Sample	Count of Total Bacteria (CFU/g)	Count of Total Fungi (CFU/g)
CK	(5.83 ± 1.28) × 10^5^ b	(1.15 ± 0.27) × 10^6^ a
UF	(5.93 ± 1.27) × 10^5^ b	(9.90 ± 0.17) × 10^5^ a
RNF	(4.87 ± 0.23) × 10^5^ b	(1.08 ± 0.37) × 10^5^ b
REF	(1.79 ± 0.97) × 10^6^ a	(7.60 ± 4.98) × 10^5^ a

Values with the same letter are not significantly different (*p* < 0.05)

#### Effects of different fertilization treatments on microbial community diversity

**Fig. 1:**
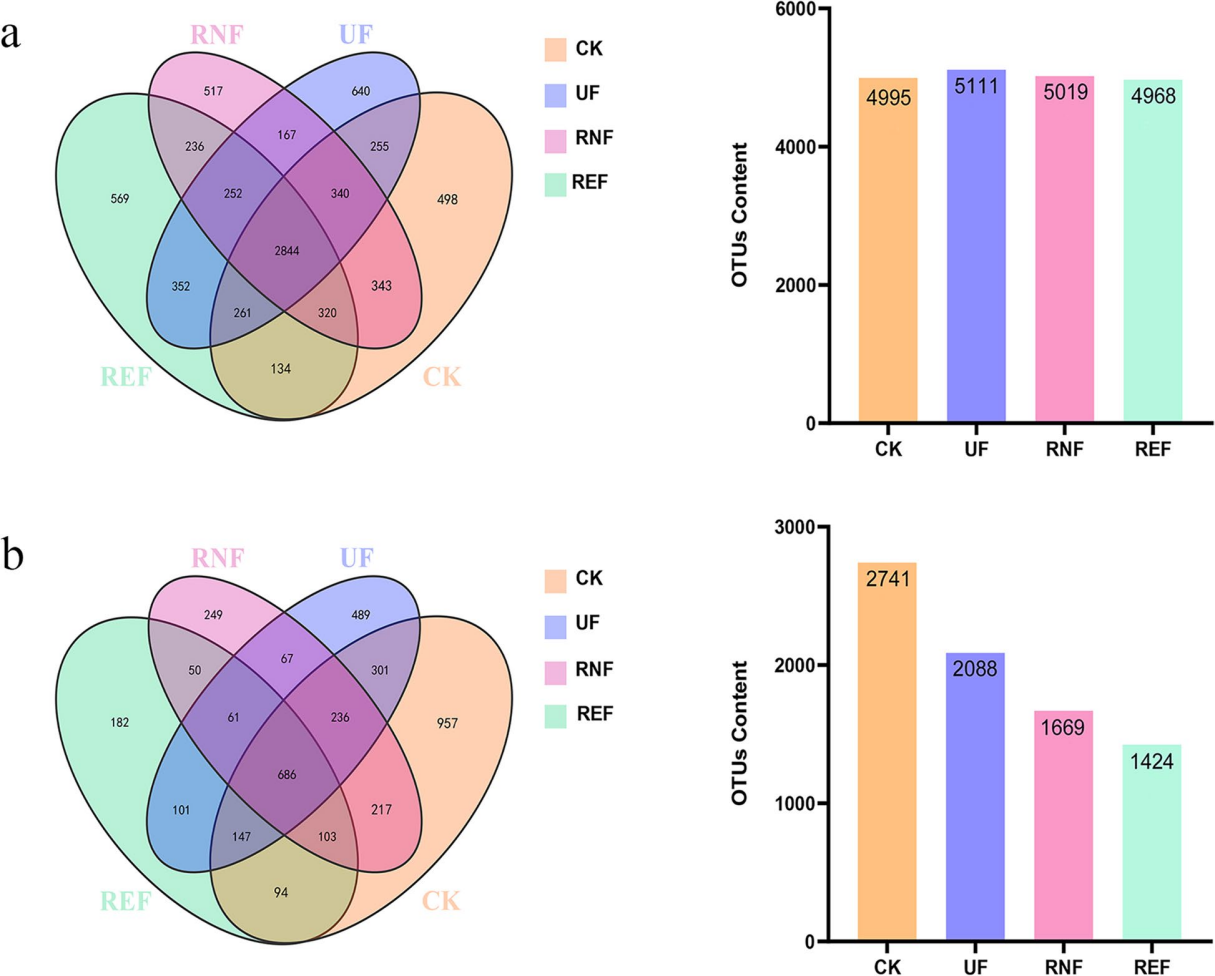
The Venn diagram of microbial communities in soils under different fertilization treatments. **a** The number of bacterial OTUs in soils under different fertilization treatments. **b** The number of fungal OTUs in soils under different fertilization treatments

In order to annotate the bacterial and fungal communities in the soils, we used classification operations to optimize the sequences. Rarefaction curve analysis showed that all four curves ended up close to flat, indicating that the sample size for sequencing was sufficient to obtain reasonable sequencing data for subsequent analysis. Rank abundance analysis showed that each curve had a large span on the horizontal axis and eventually tended to be flat, indicating that the sequenced samples were rich and species were evenly distributed (Figure S1). With a 97% uniformity threshold, there were 7735 and 3948 operational taxonomic units (OTUs) in bacterial and fungal communities. In bacterial communities, the number of OTUs in REF was slightly lower than that in other treatments, but there was no significant difference. In the fungal communities, the number of OTUs in REF was significantly lower than that in CK, UF, and RNF (Fig. 1).

In order to estimate the diversity and richness of microbial and fungal communities of the soils under four treatments, we analyzed the indices of alpha diversity (Shannon, Simpson, Chao1, and ACE) using the random sampling method (Table 2). The coverage indexes of the soil samples were greater than 0.98, indicating that the sequencing results were usable. Analysis of variance showed that the different fertilization treatments affected the bacterial and fungal communities. In the bacterial communities, the diversity index of REF was significantly lower than CK and RNF, and slightly higher than UF, but the richness index of the four treatments had no significant difference. In the fungal communities, the diversity index and richness index of REF were significantly lower than those of other treatments, as shown in Simpson and Chao1 indexes.

**Table 2:** The alpha diversity index of soils under different fertilization treatments

Sample	Shannon		Simpson		Chao1		Ace		Coverage	
	Bacteria	Fungi	Bacteria	Fungi	Bacteria	Fungi	Bacteria	Fungi	Bacteria	Fungi
CK	9.74 ± 0.12a	6.48 ± 0.54a	0.996 ± 0.001a	0.945 ± 0.034a	3710.77 ± 238.05a	1699.02 ± 174.13a	3760.11 ± 253.19a	1722.99 ± 172.11a	0.990	0.997
UF	9.19 ± 0.15b	4.48 ± 0.69b	0.992 ± 0.002c	0.748 ± 0.104b	3582.33 ± 362.49a	1288.79 ± 64.17b	3644.62 ± 373.26a	1319.68 ± 76.75b	0.989	0.997
RNF	9.55 ± 0.29ab	4.66 ± 0.28b	0.995 ± 0.001ab	0.844 ± 0.077ab	3589.52 ± 470.19a	1016.36 ± 184.40bc	3631.87 ± 489.90a	1021.08 ± 189.91c	0.990	9.998
REF	9.26 ± 0.22b	3.79 ± 0.92b	0.994 ± 0.001b	0.727 ± 0.137b	4186.13 ± 706.81a	848.48 ± 52.99c	3749.92 ± 850.96a	872.72 ± 52.12c	0.987	0.997

Values with the same letter are not significantly different (*p* < 0.05)

To further study the effects of different fertilization treatments on the distribution of soil microbial communities, beta diversity was calculated using non-metric multidimensional scaling (NMDS) and principal coordinate analysis (PCoA) (Figure S2). The results showed that soil microbial communities were different among the four fertilization treatments. To further clarify the previous separation mode of microbial community, PCoA analysis was conducted based on unweighted unifrac distance. The results showed that the confidence intervals of the four treatments were in four different quadrants. These indicated that the composition of soil microbial community was affected by different fertilization treatments.

#### Effects of different fertilization treatments on microbial community composition

**Fig. 2:**
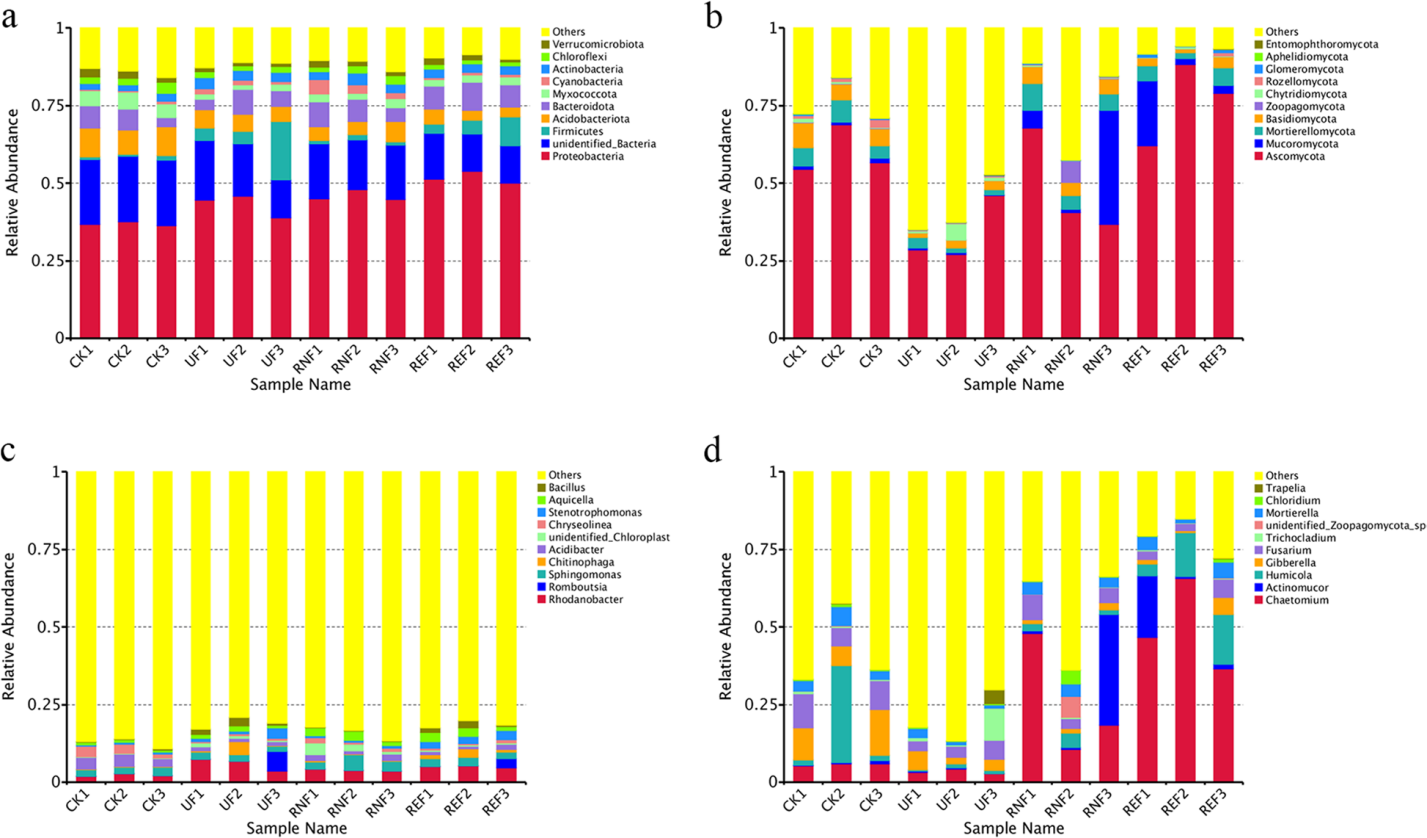
The composition of microbial communities in soils under different fertilization treatments. **a** The histogram of bacterial composition in soils under different fertilization treatments at the phylum level. **b** The histogram of fungal composition in soils under different fertilization treatments at the phylum level. **c** The histogram of bacterial composition soils under different fertilization treatments at the genus level. **d** The histogram of fungal composition in soils under different fertilization treatments at the genus level

To analyze the composition of the rhizosphere soil microbial community, the top 10 species were selected with the highest abundance at the phylum and genus levels according to the species annotation results. At the phylum level, the advantageous bacteria were Proteobacteria, Firmicutes, Acidobacter, and Bacteroidota; the advantageous fungi were Ascomycota, Mucoromycota, Mortierellomycota, and Basidiomycota. At the genus level, the advantageous bacteria were mainly *Rhodobacter*, *Romboutsia*, *Sphingomonas*, *Chitinophaga*, and *Acidibacter*. The advantageous fungi were *Chaetomium*, *Actinomucor*, *Humicola*, *Gibberella*, and *Fusarium* (Fig. 2).

**Fig. 3:**
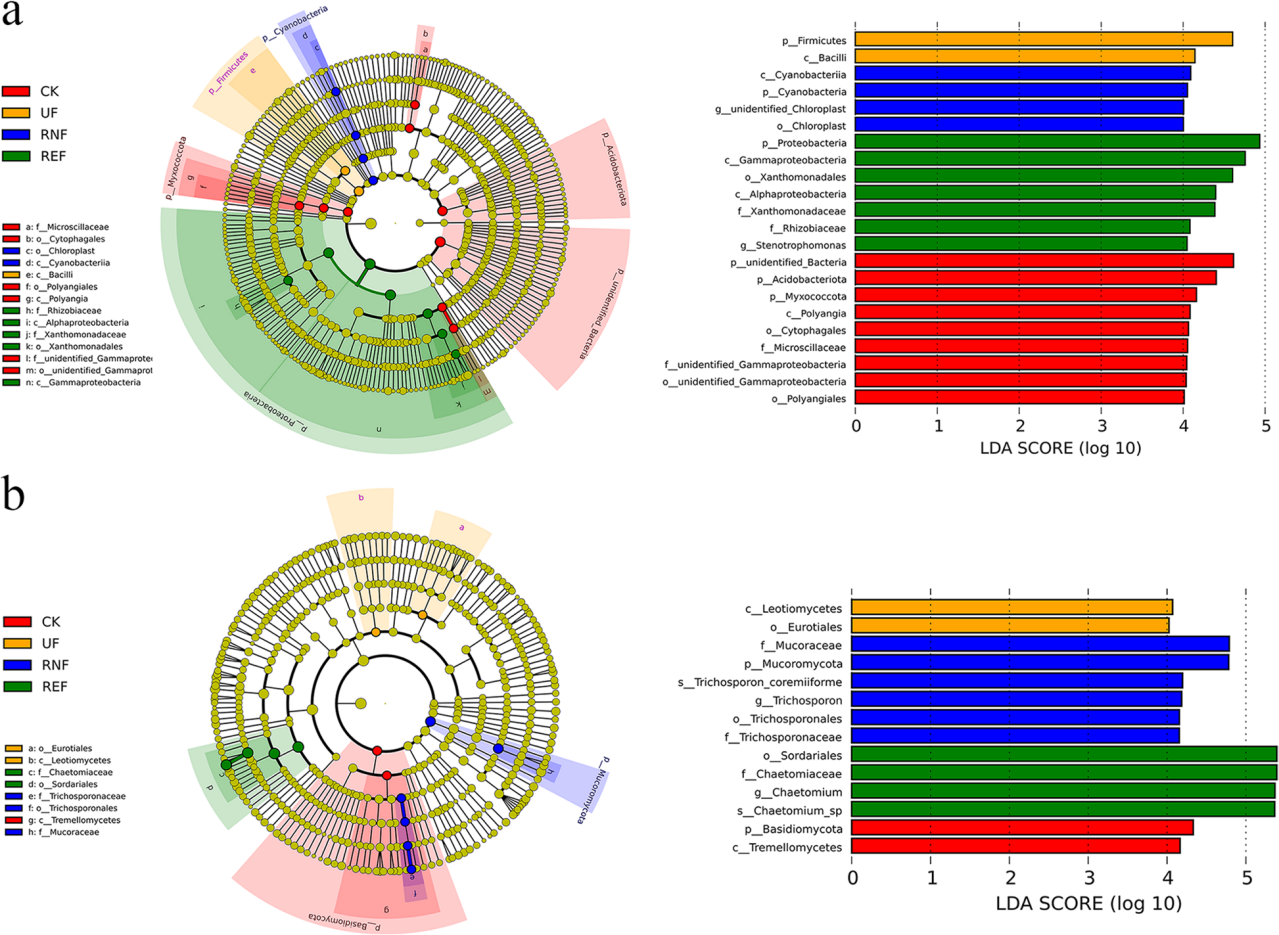
The LEfSe analysis of microbial communities in soils under different fertilization treatments. **a** The LEfSe analysis of bacterial communities in soils under different fertilization treatments. **b** The LEfSe analysis of fungal communities in soils under different fertilization treatments

To explore the bacterial and fungal communities that differed significantly differences in the soils between the four treatments, we used the LEfSe tool to analyze the microbial communities at different levels (Fig. 3). *Gammaproteobacteria* (the class to genus), *Alphaproteobacteria* (class and the family *Rhizobiaceae*), and *Sordariales* (the class to species) were significantly enriched in REF. *Cyanobacteriia* (the phylum to order, and the genus *unidentified_Chloroplast*), *Trichosporonales* (the order to species), and *Mucoromycota* (the phylum and the family *Mucoraceae*) were significantly enriched in RNF. The results showed that the bacterial and fungal communities in the soil were significantly different under different treatments.

**Fig. 4:**
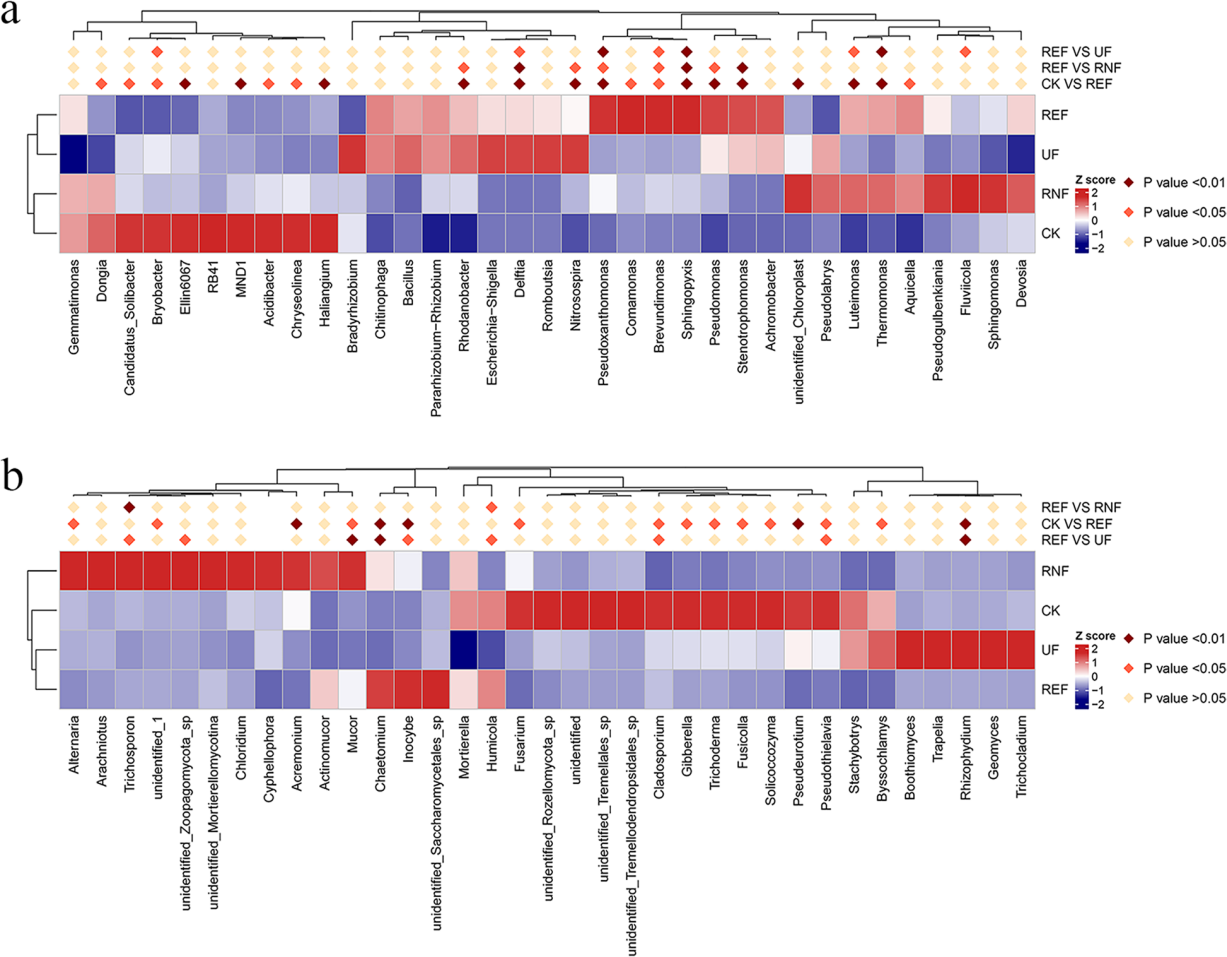
The MetaStat heatmap of microbial communities in soils under different fertilization treatments. **a** The MetaStat heatmap of bacterial communities in soils under different fertilization treatments. **b** The MetaStat heatmap of fungal communities in soils under different fertilization treatments

To further confirm the difference of microbial community composition at the genus level, MetaStat method was used to screen the species with significant differences and drawn a heat map (Fig. 4). The results showed that there were significant differences between REF and other treatments in bacterial and fungal communities. In the bacterial communities, the relative abundance of *Rhodanobacter*, *Sphingomonas*, *Delftia*, and *Nitrosospira* in REF was significantly higher than CK (*p* < 0.01); the relative abundance of *Pseudoxanthomonas*, *Sphingomonas*, and *Thermomonas* was significantly higher than that of UF (*p* < 0.01); the relative abundance of *Sphingomonas*, *Delftia*, *Stenotrophomonas* was significantly higher than that of RNF (*p* < 0.01) (Fig. 4A). In the fungal communities, the relative abundance of *Chaetomium* and *Inocybe* was significantly higher than CK (*p* < 0.01); the relative abundance of *Mucor* and *Chaetomium* was significantly higher than UF (*p* < 0.01); the relative abundance of *Trichosporon* was significantly higher than RNF (*p* < 0.01) (Fig. 4B).

#### Relationship between dominant genera of microbial communities with soil physical and chemical properties

To appraise the physical and chemical characteristics of soil under different fertilization treatments, we analyzed the nutrient contents of soil (Table 3). The results showed that the pH value was UF < REF < RNF < CK. The contents of OM, TN, TP, and AN in REF and RNF were significantly higher than those in CK and UF (*p* < 0.05), and slightly higher in REF than in RNF, while the difference was not significant (*p* > 0.05). The content of AP in REF is significantly higher than that of CK, UF, and RNF (*p* < 0.05). In addition, we measured the activities of four enzymes in the soil (Table 4). The results showed that the activities of S-UE, S-CAT, S-ACP, and S-SC in REF were significantly higher than those in CK, UF, and RNF (*p* < 0.05).

**Table 3:** The physicochemical properties of soils under different fertilization treatments

Sample	pH	OM g/kg	TN g/kg	TP g/kg	TK g/kg	AN mg/kg	AP mg/kg	AK mg/kg
CK	5.94 ± 0.10d	41.93 ± 2.79b	1.41 ± 0.91b	0.49 ± 0.10b	13.06 ± 1.57a	235.66 ± 8.08c	13.43 ± 1.21c	38.78 ± 10.46a
UF	4.63 ± 0.03a	46.11 ± 7.08b	1.59 ± 0.10ab	0.47 ± 0.08b	13.37 ± 0.55a	277.66 ± 8.08b	13.09 ± 1.03c	44.16 ± 2.56a
RNF	5.51 ± 0.12c	53.33 ± 2.20a	1.79 ± 0.22a	0.64 ± 0.23a	12.68 ± 0.28a	336.00 ± 12.12a	26.16 ± 3.50b	47.11 ± 4.70a
REF	5.15 ± 0.29b	50.07 ± 9.35a	1.79 ± 0.15a	0.68 ± 0.11a	12.75 ± 1.34a	350.00 ± 12.12a	39.35 ± 4.69a	51.95 ± 5.89a

Values with the same letter are not significantly different (*p* < 0.05)

**Table 4:** The activities of some enzymes in soils under different fertilization treatments

Sample	S-UE (μg/d/g)	S-CAT (μmol/h/g)	S-ACP (nmol/h/g)	S-SC (mg/d/g)
CK	161.45 ± 10.02c	299.86 ± 29.83ab	374.19 ± 50.64c	6.63 ± 1.11c
UF	123.03 ± 0.15c	241.27 ± 56.33d	624.05 ± 90.82b	8.42 ± 0.17bc
RNF	267.10 ± 64.99b	267.27 ± 33.33c	688.02 ± 3.43ab	10.65 ± 2.32b
REF	408.47 ± 49.23a	306.68 ± 29.53a	756.63 ± 42.83a	15.17 ± 2.26a

Values with the same letter are not significantly different (*p* < 0.05)

**Fig. 5:**
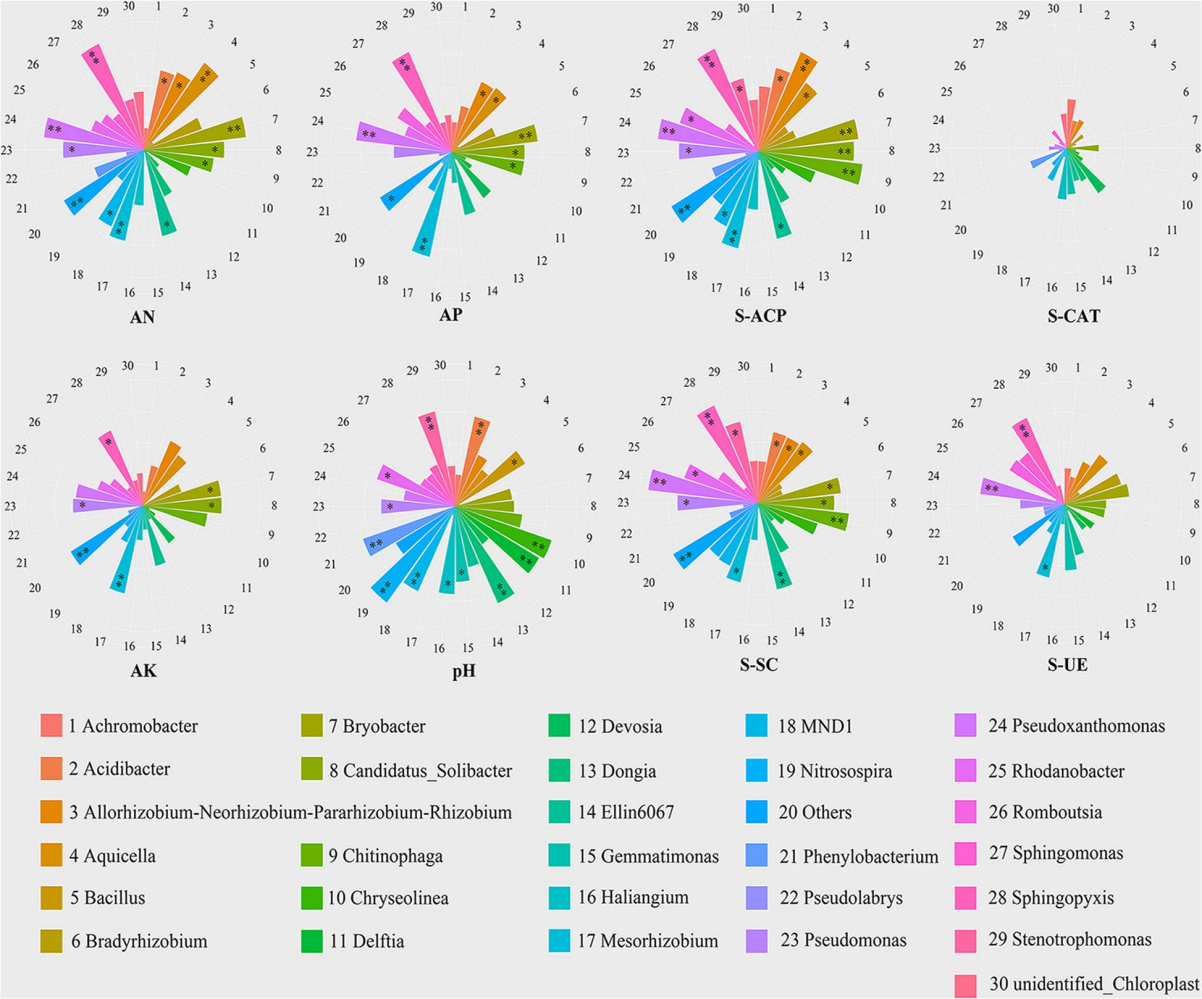
The rose diagrams of relationships between soil key properties and bacterial communities in soils with enzymatic fermented rapeseed cake. *: *p* < 0.05, **: *p* < 0.01, ***: *p* < 0.001

To investigate the effects of enzymatic rapeseed cake on soil bacterial and fungal communities, spearman correlation was used to analyze the relationship between dominant genera and major soil characteristics. Notably, AN was appreciably associated with *Aquicella*, *Stenotrophomonas*, *Trichoderma*, etc. (*p* < 0.01). AP was appreciably associated with *Mesorhizobium*, *Stenotrophomonas*, *Mucor*, *Stachybotrys*, etc. (*p* < 0.01). AK was appreciably associated with *Mesorhizobium*, *Acremonium*, and *mucor* (*p* < 0.01). PH was appreciably associated with *Dongia*, *MND1*, *Nitrosospira*, and *Phenylobacterium* (*p* < 0.01). In addition, S-ACP was appreciably associated with *Allorhizobium-Neorhizobium-Pararhizobium-Rhizobium*, *Sphingopyxis, Acremonium*, etc. (*p* < 0.01). S-SC was appreciably associated with *Pseudoxanthomonas*, *Sphingopyxis*, *Acremonium*, etc. (*p* < 0.01). S-UE was appreciably associated with *Pseudoxanthomonas*, *Sphingopyxis*, *Chaetomium*, etc. (*p* < 0.01) (Fig. 5, S3).

### Effects of different fertilization treatments on soil metabolites

#### Analysis of soil metabolites under different fertilization treatments

**Fig. 6:**
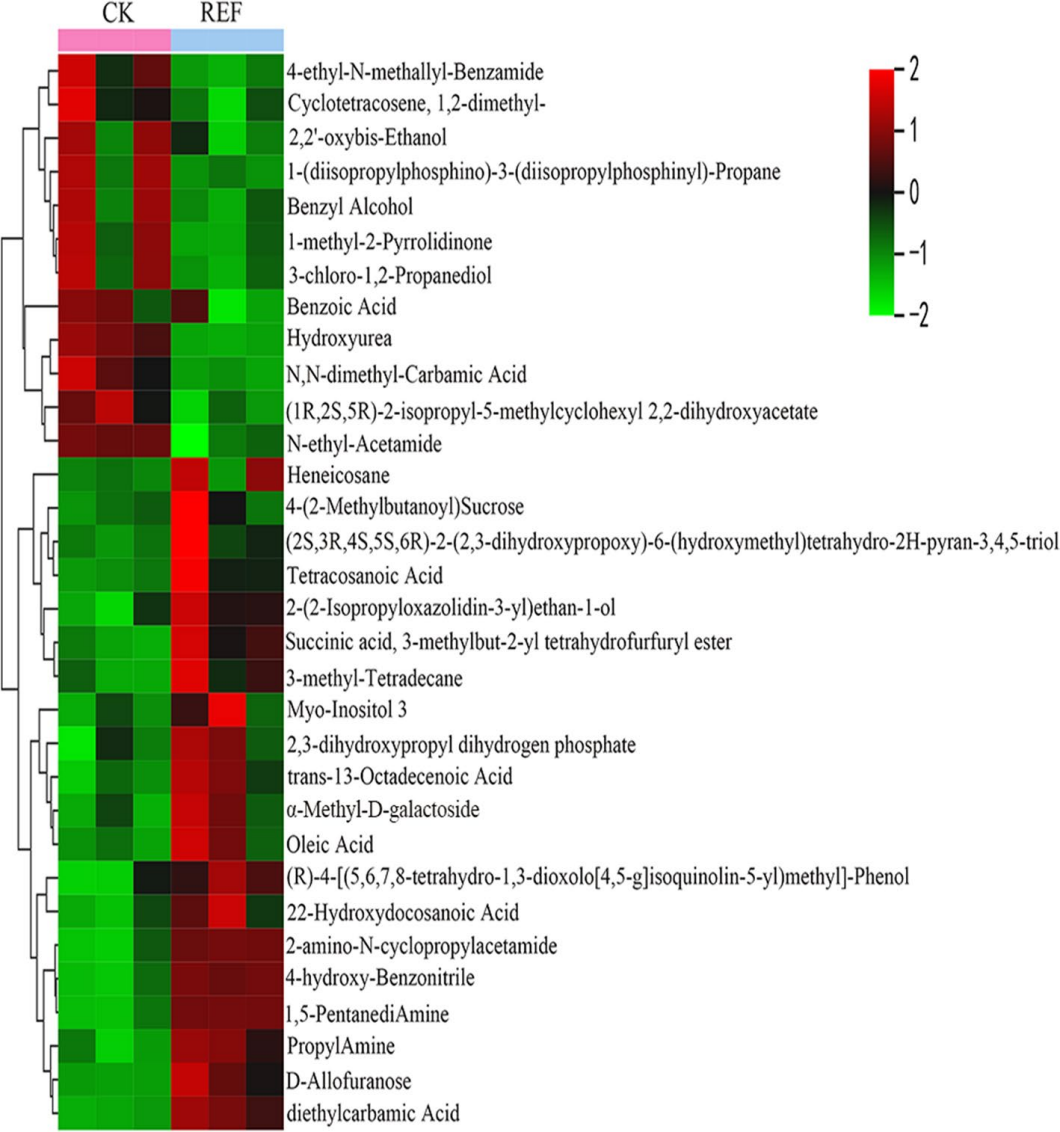
The heatmap of the differential relative content of common metabolites in CK vs REF

In order to further analyze the metabolites in soil under different fertilization treatments, we analyzed 12 soil samples on the GC–MS platform. 135 compounds were detected and quantified in soil samples, mainly including lipids (20.1%), acids (19.3%), sugars (12.6%), alcohols (12.6%), and amines (8.1%) (Figure S4A). We conducted OPLS-DA analysis on soil metabolites after different fertilization treatments to maximize the difference between REF and other treatments (Figure S4B-D). The results showed that REF was located on the positive or negative sides of the x-axis with the other three treatments and was completely separated. This indicated that the application of enzymatic rapeseed cake could significantly alter the metabolic profiling of soil. Fold_change value (≥ 1.2, ≤ 0.7) and VIP value (≥ 1) were used to screen out metabolites with significant differences, and a heatmap with hierarchical clustering analysis (HCA) was used to show metabolites differentially expressed between REF with CK, UF, and RNF. The results showed that there were 32, 47, and 24 different metabolites were screened compared with CK, UF, and RNF (Table S1-S3). REF mainly promoted the accumulation of tetracosanoic acid, 22-Hydroxydocosanoic acid, trans-13-Octadecenoic acid, oleic acid, 2,3-dihydroxypropyl dihydrogen phosphate, 4-(2-Methylbutanoyl) Sucrose, α-Methyl-D-galactoside, D-Allofuranose, heneicosane, succinic acid and so on (Fig. 6, S5). It indicated that the application of enzymatic rapeseed cake significantly stimulated the changes of rhizosphere soil metabolism, promoted the accumulation of sugar, organic acids, and lipids in the soil, and provided more beneficial and small molecular substances for the growth of tea plants.

#### Analysis of soil metabolic pathway under different fertilization treatments

**Fig. 7:**
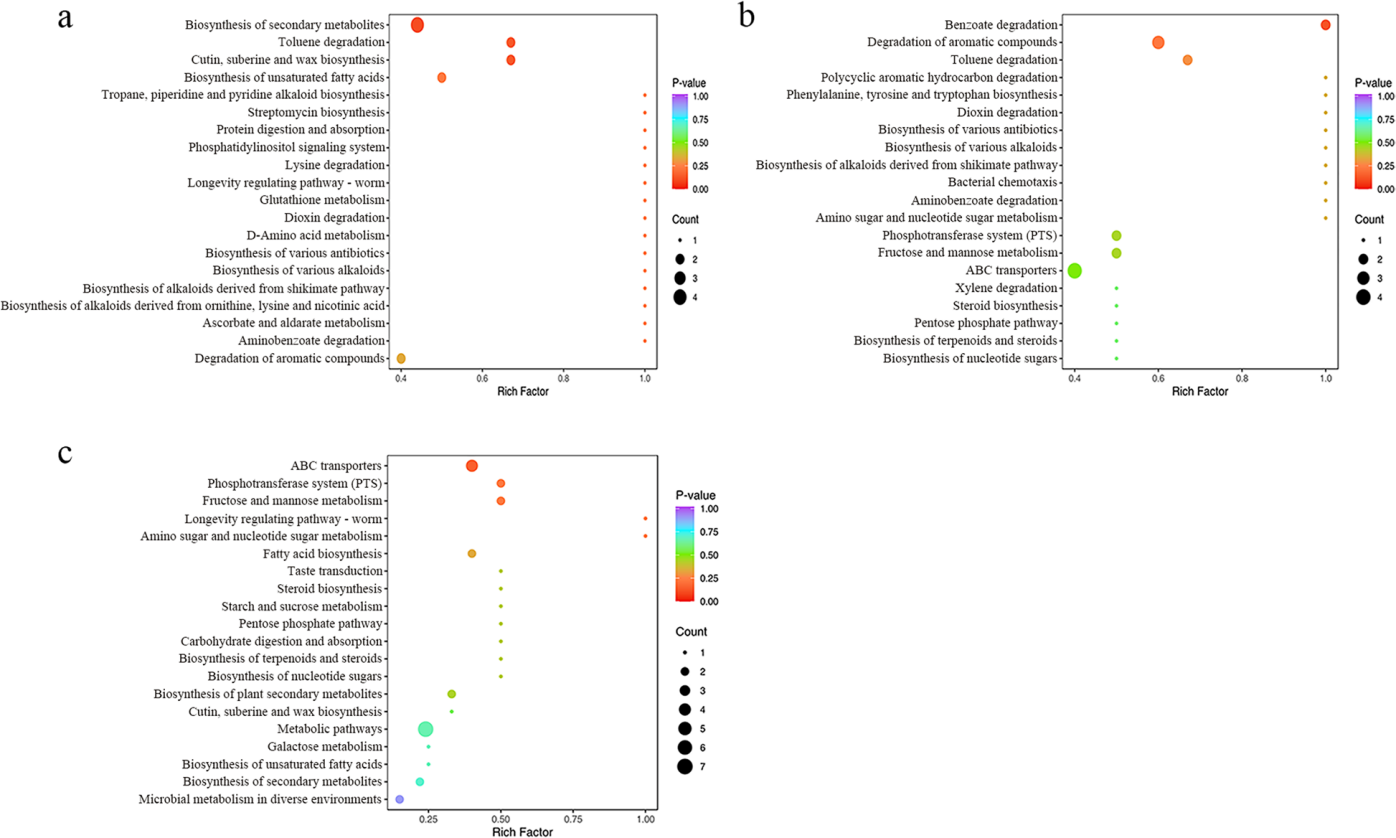
The KEGG enrichment pathway of different metabolites in soils under different fertilization treatments. **a** The KEGG enrichment pathway of different metabolites in CK vs REF group. **b** The KEGG enrichment pathway of different metabolites in UF vs REF group. **c** The KEGG enrichment pathway of different metabolites in RNF vs REF

To further analyze the role of metabolites in rhizosphere soil under different fertilization treatments, we enriched the differential compounds through the Kyoto Encyclopedia of Genes and Genomes (KEGG) pathway. The results showed that, compared REF with CK, the differential metabolites were mainly concentrated in the biosynthesis of unsaturated fatty acids, biosynthesis of secondary metabolites, toluene degradation, and the biosynthesis of cutin, suberine and wax (Fig. 7A). Compared REF with UF, the differential metabolites were mainly concentrated in the benzoate degradation, degradation of aromatic compounds, and toluene degradation (Fig. 7B). Compared REF with RNF, the differential metabolites were mainly concentrated in the ABC transporters, phosphotransferase system (PTS), fructose and mannose metabolism, amino sugar, and nucleotide sugar metabolism (Fig. 7C). In particular, compared with other treatments, the differential enrichment pathways of REF were sugar and fatty acid metabolisms.

### Correlation between microorganisms and metabolites under different fertilizer treatments

**Fig. 8:**
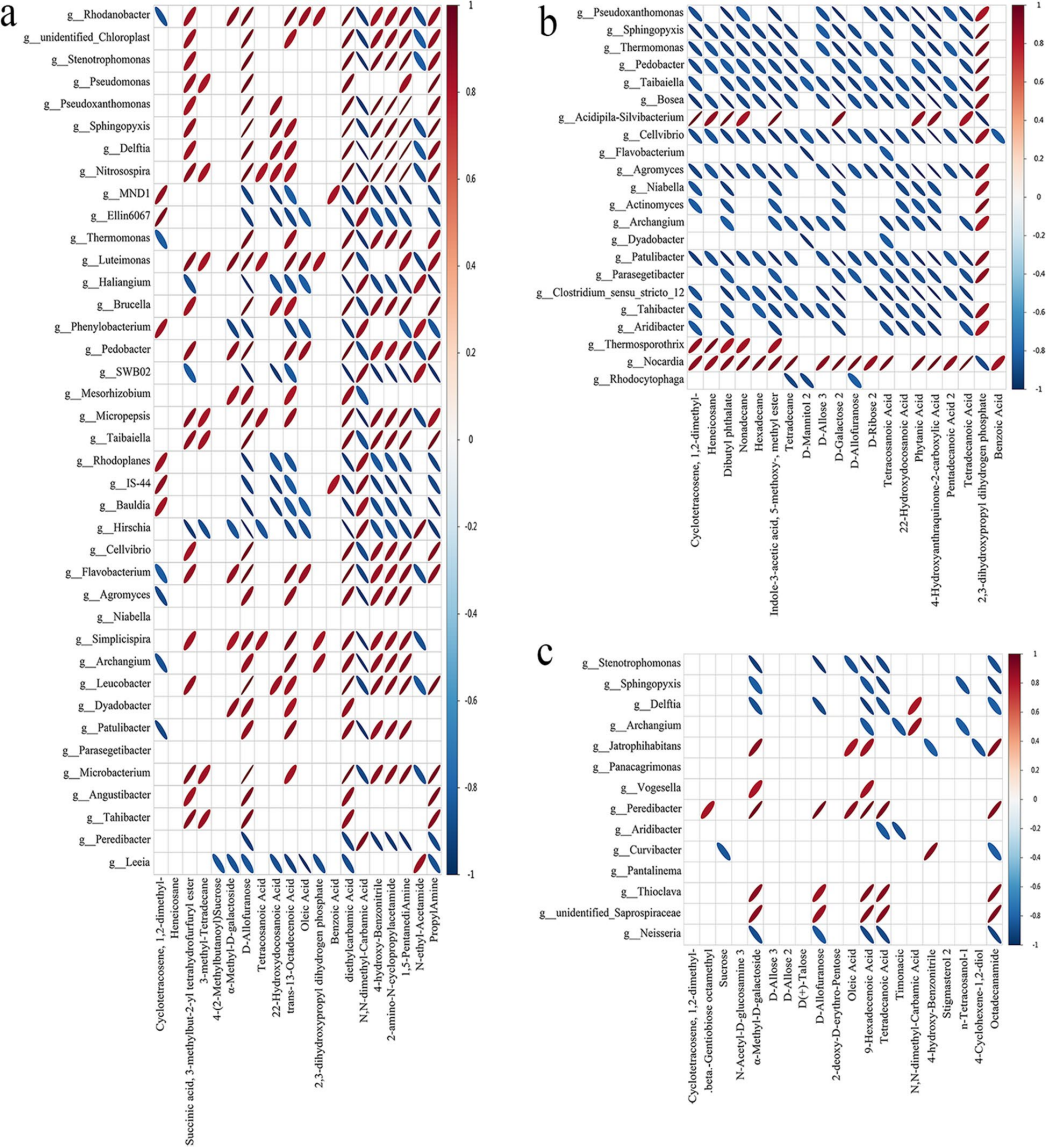
The ellipse heatmap of relationships between bacterial communities and soil metabolites. **a** The relationship of bacterial communities and soil metabolites in CK vs REF. **b** The relationship of bacterial communities and soil metabolites in UF vs REF. **c** The relationship of bacterial communities and soil metabolites in RNF vs REF

To study the correlation between microorganisms and metabolites in soil, the spearman coefficient was used for analysis. Correlation heatmap was used to show the correlation between significantly different microbial populations and metabolites (R^2^ ≥ 0.64, *p* < 0.05). In the bacterial communities, REF compared to CK, organic acids (trans-13-Octadecenoic acid, diethylcarbamic acid, oleic acid, and tetracosanoic acid) were appreciably positively correlated to *Luteimonas*, *Rhodanobacter*, *Nitrosospira*, *Delftia*, *Sphingopyxis*, *Brucella*, etc. Carbohydrates (D-Allofuranose and α-Methyl-D-galactoside) were appreciably positively correlated to *Rhodanobacter*, *Luteimonas*, *Pedobacter*, *Flavobacterium*, and *Dyadobacter*. Lipids (succinic acid, 3-methylbut-2-yl tetrahydrofurfuryl ester, and 3-methyl-Tetradecane) were appreciably positively correlated to *Tahibacte*, *Pseudomonas*, *Nitrosospira*, *Micropepsis*, *Taibaiella*, and *Microbacterium* (Fig. 8A). REF compared to UF, organic acids (phytanic Acid, 4-Hydroxyanthraquinone-2-carboxylic acid, and tetradecanoic acid) were appreciably positively correlated to *Nocardia* and *Acidipila-Silvibacterium*. Carbohydrates (D-Allose 3, D-Galactose 2, D-Allofuranose, and D-Ribose 2) were significantly positively correlated to *Nocardia*. Lipids (nonadecane, methyl ester, heneicosane, and dibutyl phthalate) were appreciably positively correlated to *Thermosporothrix*, *Nocardia*, and *Acidipila-Silvibacterium* (Fig. 8B). REF compared to RNF, α-Methyl-D-galactoside, D-Allofuranose, 9-Hexadecenoic acid, tetradecanoic acid, and octadecanamide were appreciably positively correlated to *Jatrophihabitans*, *Peredibacter*, and *Thioclava* (Fig. 8C).

In the fungal communities, REF compared to CK, *Chaetomium* was appreciably positively correlated to α-Methyl-D-galactoside, D-Allofuranose, diethylcarbamic acid, PropylAmine, and 1,5-PentanediAmine. *Cintractia*, *Geopora*, *Lecophagus*, and *Pleotrichocladium* were appreciably positively correlated to N,N-dimethyl-Carbamic acid. *Byssochlamys*, *Pseudeurotium*, and *Paraphaeosphaeria* were appreciably positively associated with Cyclotetracosene, 1,2-dimethyl- (Figure S6A). REF compared to UF, *Sagenomella* and *Byssochlamys* were appreciably positively correlated to lipids (heneicosane, dibutyl phthalate, and nonadecane). *Paracremonium*, *Sagenomella*, *Byssochlamys*, and *Pleotrichocladium* were appreciably positively correlated to D-Galactose 2, phytanic acid, and tetradecanoic acid (Figure S6B). REF compared to RNF, *Trichosporon*, *Setophaeosphaeria*, and *Exophiala* were appreciably positively correlated to α-Methyl-D-galactoside, D-Allofuranose, 9-Hexadecenoic acid, tetradecanoic acid, n-Tetracosanol-1, and octadecanamide. *Trichosporon* was appreciably positively correlated to stigmasterol2 and timonacic. *Exophiala* was appreciably positively correlated to D-Allose 3, stigmasterol2, and oleic acid (Figure S6C).

## Discussion

### The relative abundance of beneficial microorganisms in the soil was increased after the application of enzymatic rapeseed cake

The diversity and composition of soil microbial community are crucial to the functioning of soil and the stability of the ecological environment [19, 20]. It was reported that the bacterial diversity after applying organic fertilizer was significantly higher than that after applying chemical fertilizer in soil of crops [21–24]. Becker and Backer et al. found that inoculation of the PGPR strain or synthetic community was usually hindered by the original community in the soil, which affected the resilience of the inoculated strain, leading to the rapid disappearance or collapse of the introduced strain [25, 26]. In this study, it was found that the bacterial diversity of tea rhizosphere soil after the application of fermented rapeseed cake was significantly lower than that of the control with no fertilization but higher than that of the urea treatment. We speculated that, on the one hand, due to the increase of beneficial microorganisms after the application of rapeseed cake, the survival of indigenous microorganisms in the soil was inhibited, and the soil microbial diversity was reduced. On the other hand, because rapeseed cake was rich in microbial communities after being applied to the soil, indigenous microorganisms hinder the vitality of the original communities in rapeseed cake, resulting in the reduced viability of some introduced microbial communities.

By dissecting the composition of rhizosphere soil microbial communities at the genus level, we found that the dominant genera of tea rhizosphere soil were mainly *Rhodanobacter*, *Sphingomonas*, *Acidibacter*, *Romboutsia*, *Chitinophaga*, *Chaetomium*, *Humicola*, *Gibberella*, *Fusarium*, and *Actinomucor* (Fig. 2C, D). Moreover, after the application of enzymatic rapeseed cake, the relative abundance of *Pseudoxanthomonas*, *Pseudomonas*, *Sphingomonas*, *Stenotrophomonas*, *Chaetomium*, and *Inocybe* in soil was higher than that of other treatments (Fig. 4). Previous studies have shown that *Pseudoxanthomonas*, *Pseudomonas*, *Rhodanobacter,* and *Sphingomonas* were bacterial groups related to the nitrogen cycle and can participate in nitrogen fixation, denitrification, and other processes to promote plant growth [27–32]. *Sphingomonas* was defined as an acid-dependent biomarker that could survive and dominate in soil with pH < 5 [33–35]. *Stenotrophomonas* was an important contributor to the mineralization of organic phosphorus. It could reduce the metal ion damage induced by low pH by secreting organic acids and producing iron carriers [36–38]. *Inocybe* was usually related to plants that form ectomycorrhizal symbionts, which could transport water and nutrients in plants [39]. *Chaetomium* was a kind of fungus beneficial to plants, which could effectively control the infection of plant pathogens [40–42]. In our study, the above types of microorganisms were found in rhizosphere soil microorganisms after the application of rapeseed cake. This indicated that the application of enzymatic rapeseed cake increased the relative abundance of beneficial microbial flora, promoted the nitrogen and phosphorus cycling in the rhizosphere of tea plants, promoted the biochemical changes in the soil, protected the roots of tea plants, and made the soil microenvironment more suitable for the growth of tea plants.

### The enzymes and microorganisms jointly promoted the transformation of nutrients after the application of enzymatic rapeseed cake in the soil

Soil enzyme activity is closely related to soil nutrient status. Ge et al. found that there was a significant correlation between organic carbon with soil invertase, and total nitrogen with soil urease respectively after long-term fertilization in northern China [43]. Ning et al. found that soil organic matter, soil catalase activity, and urease activity increased significantly after applying organic in vegetable fields [44]. Zhang et al. found that the activities of soil sucrase, soil acid phosphatase, and soil urease were significantly higher than those of inorganic fertilizer after applying soybean fertilizer in the tea garden [10]. In this study, it was found that after the application of enzymatic rapeseed cake, the content of soil organic matter (OM), total nitrogen (TN), total phosphorus (TP), available nitrogen (AN), and available phosphorus (AP) was significantly higher than that of the control with no fertilization and urea treatment, and slightly higher than that of the naturally fermented rapeseed cake treatment. The activities of soil urease (S-UE), soil catalase (S-CAT), soil acid phosphatase (S-ACP), and soil invertase (S-SC) were also significantly higher than those of the control with no fertilization and urea treatment, and slightly higher than those of the naturally fermented rapeseed cake treatment. This showed that the application of enzymatic rapeseed cake could improve soil nutrients, activate soil enzyme activity, reduce soil pH value, and thus help to improve the quality of tea garden soil.

Microbial growth depended on soil nutrients and was affected by changes in soil nutrients [45–47]. Zhalnina and Naz et al. found that soil pH was the key factor of bacterial diversity, which had a strong impact on soil microbial community and was a strong driving force for microbial growth and community composition [48, 49]. Li and Duff et al. found that the application of fertilizer in soil changed of microbial functional diversity changed with nitrogen content [50, 51]. Ding et al. found that the application of organic manure in crop soil significantly correlated the microbial community with soil pH, available phosphorus, and total phosphorus concentration [52]. In this study, the spearman correlation coefficient was used to evaluate the relationship between the physical and chemical properties of tea soil and the microbial community at the genus level. It was found that pH, AN, and AP were the main environmental factors affecting tea soil microorganisms, and were significantly positively correlated with the main beneficial microorganisms (Fig. 5, S3). *Dongia* was significantly positively correlated with pH. *Aquicella*, *Pseudoxanthomonas*, and *Sphingpopyxis* were significantly positively correlated with AN. *Mesorhizobium*, *Sphingopyxis*, and *Chaetomium* were significantly positively correlated with AP (R ≥ 0.8, p ≤ 0.01). In addition, *Chaetomium* was significantly positively correlated with S-UE. *Chitinophaga*, *Pseudoxanthomonas*, and *Sphingopyxis* were significantly positively correlated with S-ACP. *Sphingopyxi* was significantly positively correlated with S-SC (R ≥ 0.8, p ≤ 0.01). These microorganisms played an important role in the metabolism of tea rhizosphere. This study is similar to our previous results in *The application of enzymatic fermented soybean effectively regulates associated microbial communities in tea soil and positively affects lipid metabolites in tea new shoots* [10]. Therefore, we inferred that it was the enzyme and microorganism jointly promoted the transformation of substances in the soil.

### The soil microorganisms further stimulated the metabolism of sugar and fatty acids after the application of enzymatic rapeseed cake

It was found that the main metabolic pathways during the enzymatic fermentation of rapeseed cake were glucosinolate biosynthesis, galactose metabolism, Aminoacyl-tRNA biosynthesis, starch and sucrose metabolism, linoleic acid metabolism, biosynthesis of secondary metabolites, and inositol phosphate metabolism. The metabolite enrichment pathways were specific in the process of enzymatic fermentation, which indicated that the metabolites of enzymatic fermentation mainly focused on sugar metabolism and fatty acid metabolism [18].

In this study, the distribution of metabolites in soil was obviously separated from other treatments after the application of enzymatic rapeseed cake (Fig. S4B-D). It was proved that the application of enzymatic rapeseed cake could further promote the metabolism of sugars (α-Methyl-D-galactoside, D-Allofuranose, etc.), organic acids (tetracosanoic acid, 22-Hydroxydocosanoic acid, trans-13-Octadecenoic acid, etc.) and lipids (Heneicosane, 3-methyl-Tetradecane, etc.) in soil (Fig. 6). The differential metabolites were mainly concentrated on the metabolic pathways of sugar and fatty acids. Sugar and lipids were the key metabolites in soil microorganisms and were crucial in the interaction between plants and rhizosphere microorganisms [53–55]. In this study, it was also found that after the application of enzymatic rapeseed cake, the number of soil microorganisms closely related to sugar and lipid substances increased significantly, and most of them were positively correlated, especially *Chaetomium*, *Rhodanobacter*, and *Luteimonas* were significantly positively correlated with sugar; *Tahibacte*, *Pseudomonas*, and *Nitrosospira* were significantly positively correlated with lipids (Fig. 8A). These results showed that the enzymatic rapeseed cake applied in the soil further stimulated the activities of microbial related to the metabolism of sugar and fatty acids. Therefore, we speculated that the beneficial microorganisms in the soil further promoted the fermentation of rapeseed cake, further increased the contents of small molecular compounds in the soil, and enhanced the nutritional effects of enzymatic rapeseed cake.

## Conclusions

In this study, the application of enzymatic rapeseed cake improved the nutrient status of tea rhizosphere soil, increased the relative abundance of key microorganisms, and further increased the contents of key metabolites in the soil, which was of great value for raising the productivity of tea garden soil. In conclusion, this study laid an important foundation for further application of enzymatic rapeseed cake as organic fertilizer in tea gardens.

## Materials and methods

### Test processing and collection

The experiment was conducted in the scientific research intelligent greenhouse of Qingdao Agricultural University, Shandong Province, China (36°18′ N, 120°07′ E) in 2021. The environmental conditions in the greenhouse were as follows: the temperature was 26 °C/20 °C (day/night), the air humidity was 50%, and the lighting time was 12 h a day. Two-year-old tea seedlings ‘*Zhongcha 108*’ were used as experimental materials [10]. Plant tea seedlings with the same growth vigor in plastic pots, and there are 3 tea seedlings in each pot. The soil was brown loam with a pH of 5.94, and soil organic matter of 41.95 g/kg. The fertilizer is rapeseed cake which has been fermented before [18].

The experiment was divided into four groups of treatments: control with no fertilizer (CK), urea fertilization (UF), naturally fermented rapeseed cake fertilization (RNF), and enzymatic rapeseed cake fertilization (REF). Each treatment consisted of 8 pots and 3 repeats. The amount of fertilizer shall be applied with equal nitrogen [56]. Soil samples were collected after 36 days of fertilization treatment. Then, each sample with 3 replications was divided into three parts, one part was stored at 4^◦^C to determine its nutritional properties, one part was frozen at -80^◦^C to determine its microbial composition and metabolites, and the other part was dried with natural air for analysis and determination.

### Determination of soil nutritional indicators

Soil pH was measured using a pH meter (PHSJ-4A, Shanghai Yidian Scientific Instrument Co., Ltd, China) in the supernatant of 1:5 soil–water mixtures. The soil organic matter (OM) was determined using the potassium dichromate oxidation with an external heating method [57, 58]. The total nitrogen (TN) was determined by the Kjeldahl method, total phosphorus (TP) was determined by the AutoAnalyzer system, and total potassium (TK) was determined by atomic absorption spectrophotometer [59, 60]. Soil alkaline nitrogen (AN) was determined by the alkaline diffusion method [61], soil available phosphorus (AP) was determined using the molybdenum blue method after extraction with sodium bicarbonate, and soil available potassium (AK) was determined by flame photometry after extraction with ammonium acetate solution [62, 63]. The activities of the soil urease (S-UE), soil catalase (S-CAT), soil acid phosphatase (S-ACP), and soil sucrase (S-SC) were measured using the S-UE kit (No. G0301W), S-CAT kit (No. G0303W), S-ACP kit (No. G0304W), and S-SC kit (No. G0302W) (Suzhou Grace Biotechnolgy Co., Ltd., Suzhou, China), respectively.

### Determination of soil microbial community quantity

Nutrient agar (NA) (HB0109, Haibo Biological, China) and potato dextrose agar (PDA) (HB0233, Haibo Biological, China) culture media were configured, sterilized, and set aside. Under aseptic conditions, 1.0 g of soil sample was weighed and 9 mL of sterilized saline was added and mixed to make a 1:10 dilution. And sequentially diluted into 1:100, 1:1000, 1:10,000, 1:100,000 dilution spare. Took 1 mL of water samples of three suitable dilutions and injected them into sterilized petri dishes, poured about 15 mL of NA and PDA culture medium cooled to about 45 °C, and immediately swirled the petri dishes to mix the water samples with the culture medium well. Allowed to cool and set, then turned the petri dishes over so that the bottom was up. NA culture medium was placed in a 37 °C incubator for 48 h, and PDA culture medium was placed in a 29 °C incubator for 72 h for colony counting. Two parallels were made for each dilution of each type of petri dish and a blank control was made. Observations were made under a microscope and colonies were counted from each petri dish. The total number of bacteria was calculated from the number of NA colonies and the total number of fungi was calculated from the number of PDA colonies.

### Determination of soil microbiomes

The method of soil DNA extraction was as described in our previous paper [18]. The DNA samples were individually amplified in V4 hyper variable regions by polymerase chain reaction (PCR) using primers 515F and 806R for 16S rDNA in bacteria, and primers ITS5-1737F and ITS2-2043R for ITS in fungi [10]. Phusion® High fidelity PCR Master Mix with GC buffer (New England Biolabs, United States) and high-efficiency and high-fidelity enzyme for PCR to ensure amplification efficiency and accuracy. PCR amplification and Illumina NovaSeq sequencing were performed according to previous methods [18].

Split each sample data from the offline data based on the Barcode sequence and PCR amplification primer sequence, cut off the Barcode and primer sequences, and used FLASH (V1.2.7, http://ccb.jhu.edu/software/FLASH/) to splice the reads of each sample [64]. The resulting splicing sequence was the raw tag data (Raw Tags); The raw tags obtained by splicing require strict filtering to obtain high-quality tag data (Clean Tags) [65]. Referring to Qiime's (V1.9.1 http://qiime.org/scripts/split_libraries_fastq.html) tags quality control process: performed tag interception, length filtering, and removal of chimeric sequences to obtain the final effective tags [66, 67]. Clustered all Effective Tags of all samples using the Uparse (V7.0.1001, http://www.drive5.com/uparse/) [68]. By default, the sequences were clustered with 97% identity to become OTUs. At the same time, representative sequences of OTUs were selected, and based on their algorithm principles, the sequence with the highest frequency of occurrence among OTUs was selected as the representative sequence of OTUs. The OTUs sequence was annotated with species, and the Mothur method and the SSUrRNA database of SILVA138 (http://www.arb-silva.de/) were used for species annotation analysis (with a threshold of 0.8 ~ 1) to obtain Taxonomy information, and the community composition of each sample was counted at each classification level: kingdom, phylum, class, order, family, genus, and species [69–71]. At a certain level of classification (phylum, order, genus, species level, or OTUs), the relative abundance of a species/OTU was the number of tags corresponding to a certain species/OTU in the sample divided by the total number of OTUs corresponding to the total clustering of the sample.

### Determination of soil metabolites by GC–MS

GC–MS analysis of soil was performed on Agilent 8890 gas chromatograph coupled to a 5977B mass spectrometer with a DB-5MS column (30 m length × 0.25 mm i.d. × 0.25 µm film thickness, J&W Scientific, USA). The GC–MS procedure was modified based on previous studies [72, 73]. Briefly, grinded the freeze-dried soil sample into powder at room temperature, weighed 500 mg of powder, and added 1 mL methanol: isopropanol: water (3:3:2 V/V/V) extract, vortexed for 3 min, and ultrasound for 20 min. The extracts were centrifuged at 12,000 r/min under 4 °C for 3 min. Then, helium was used as carrier gas, at a flow rate of 1.2 mL/min. Injections were made in the front inlet mode with a split ratio of 5:1, and the injection volume was 1 μL. The oven temperature was held at 40 °C for 1 min, and then raised to 100 °C at 20 °C/min, raised to 300 °C at 15 °C/min, and held at 300 °C for 5 min. All samples were analyzed in scan mode. The ion source and transfer line temperature were 230 °C and 280 °C, respectively. Finally, quality control and data annotation were performed. Soil metabonomic analysis was conducted by MetWare Biotechnology Co., Ltd (Wuhan, China).

### Statistical analysis

Statistical analysis was performed using SPSS 25.0 software (SPSS Inc., Chicago, United States). One-way analysis of variance (ANOVA) and Duncan’s test were used to determine significant differences (*p* < 0.05) among soil properties and enzyme activities, as well as the relative abundance of microbial taxa. The alpha diversity index was calculated with R (Version 1.9.1), and the beta diversity index between groups was analyzed with R (Version 2.15.3). PCoA analysis used the WGCNA, stats, and ggplot2 software packages of R (Version 2.15.3), and NMDS analysis used the vegan software package of R (Version 2.15.3). LEfSe analysis used LEfSe software, and the default screening value of the LDA Score was set to 4. Metastats analysis performed permutation test between groups at each classification level using R software to obtain the *P* value, and then modified the *P* value by Benjamini and Hochberg False Discovery Rate method to obtain the Q value [74]. The relationship between rhizosphere microbial community structure and soil environmental factors, as well as the relationship between rhizosphere microbial community structure and soil metabolites, were analyzed by Spearman correlation using the pheatmap software package [75].

The HCA (hierarchical cluster analysis) results of samples and metabolites were presented as heatmaps with dendrograms, while Pearson correlation coefficients (PCC) between samples were calculated by the core function in R and presented as only heatmaps [76]. Both HCA and PCC were carried out by R package ComplexHeatmap. For HCA, normalized signal intensities of metabolites (unit variance scaling) are visualized as a color spectrum. Significantly regulated metabolites between groups were determined by VIP ≥ 1 and absolute log2FC (fold change) ≥ 1. VIP values were extracted from OPLS-DA result, which also contains score plots and permutation plots, and were generated using the R package MetaboAnalystR [77, 78]. The data was log transform (log2) and mean centering before OPLS-DA. In order to avoid overfitting, a permutation test (200 permutations) was performed. Identified metabolites were annotated using the KEGG Compound database (http://www.kegg.jp/kegg/compound/), and annotated metabolites were then mapped to the KEGG Pathway database (http://www.kegg.jp/kegg/pathway.html). Pathways with significantly regulated metabolites mapped to were then fed into MSEA (metabolite sets enrichment analysis), and their significance was determined by the hypergeometric test’s *p*-values [79].

### Supplementary Information

**Additional file 1: Figure S1.** The Rarefaction curve and Rank abundance of microbial communities in soils under different fertilization treatments. **Figure S2.** The NMDS and PCoA analysis of microbial communities in soils under different fertilization treatments. **Figure S3.** The rose diagrams of relationships between soil key properties and fungal communities in soils with enzymatic fermented rapeseed cake. **Figure S4.** (a) The pie chart of soil metabolite composition. (b) The OPLS-DA analysis of metabolites in CK vs REF. (c) The OPLS-DA analysis of metabolites in UF vs REF. (d) The OPLS-DA analysis of metabolites in RNF vs REF. **Figure S5.** (a) The heatmap of the differential relative content of common metabolites in UF vs REF. (b) The heatmap of the differential relative content of common metabolites in RNF vs REF. **Figure S6.** The ellipse heatmap of relationships between fungal communities and soil metabolites.

**Additional file 2: Table S1.** Differential metabolite in CK vs REF. **Table S2.** Differential metabolite in UF vs REF. **Table S3.** Differential metabolite in RNF vs REF.

## Data Availability

The raw sequencing data were deposited in NCBI Sequence Read Archive (SRA) under accession number PRJNA887563 for bacteria and PRJNA887678 for fungi.

## Abbreviations

CK: Control with no fertilizer
UF: Urea fertilization
RNF: Naturally fermented rapeseed cake fertilization
REF: Enzymatic rapeseed cake fertilization
CFUs: Colony-forming units
OTUs: Operational taxonomic units
NMDS: Non-metric multi-dimensional scaling
PCoA: Principal coordinate analysis
OM: Organic matter
TN: Total nitrogen
TP: Total phosphorus
TK: Total potassium
AN: Available nitrogen
AP: Available phosphorus
AK: Available potassium
S-UE: Soil urease
S-CAT: Soil catalase
S-ACP: Soil acid phosphatase
S-SC: Soil sucrase
