# Supplementary material for: Enzymatic fermentation of rapeseed cake significantly improved the soil environment of tea rhizosphere
Source: BMC Microbiol. 2023 Sep 7;23:250. doi: 10.1186/s12866-023-02995-7 (PMC10483718; doi:10.1186/s12866-023-02995-7)

Figure S1 The Rarefaction curve and Rank abundance of microbial communities in soils under different fertilization treatments.

(a) The Rarefaction curve of bacterial composition in soils under different fertilization treatments. (b) The Rarefaction curve of fungal composition in soils under different fertilization treatments. (c) The Rank abundance of bacterial composition in soils under different fertilization treatments. (d) The Rank abundance of fungal composition in soils under different fertilization treatments.

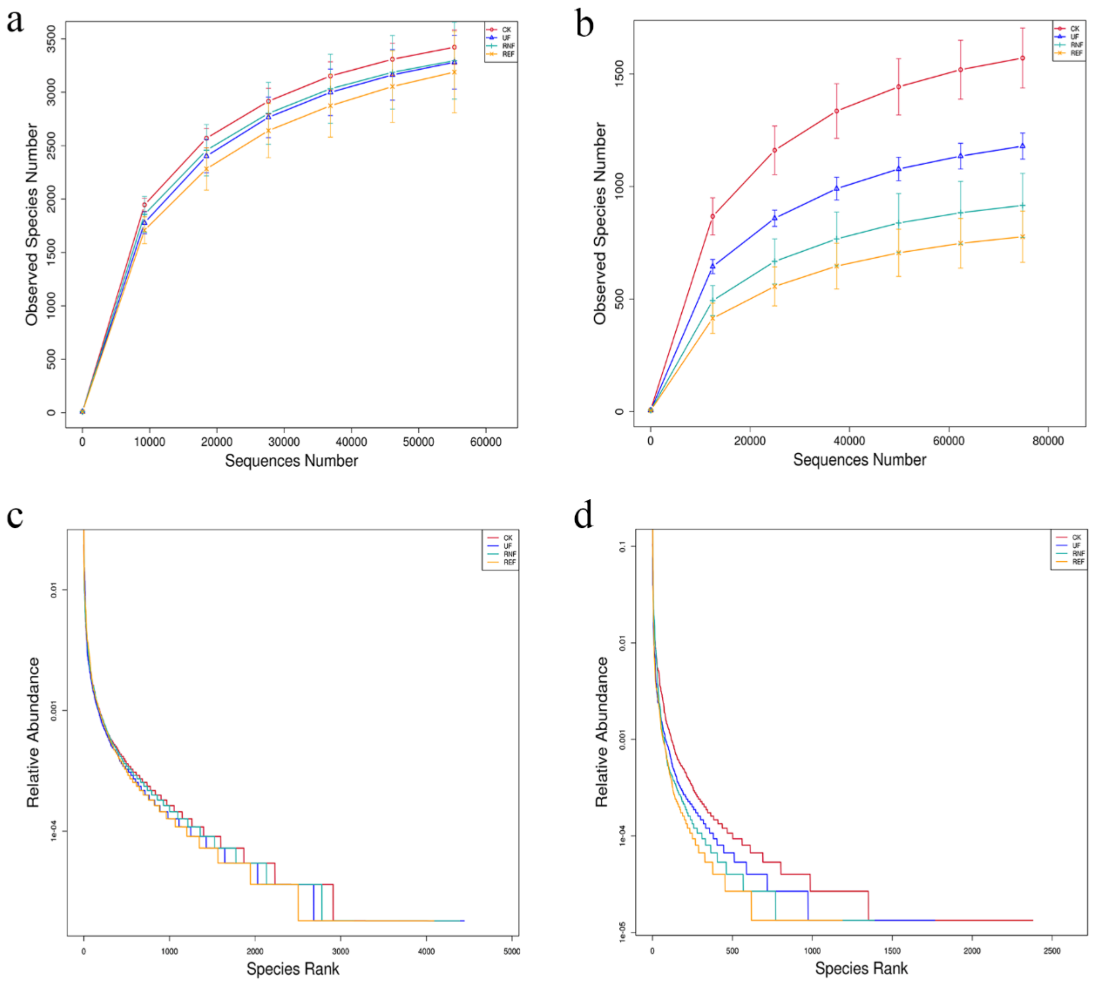

Figure S2 The NMDS and PCoA analysis of microbial communities in soils under different fertilization treatments.

(a) The NMDS analysis of bacterial composition in soils under different fertilization treatments. (b) The NMDS analysis of fungal composition in soils under different fertilization treatments. (c) The PCoA analysis of bacterial composition in soils under different fertilization treatments. (d) The PCoA analysis of fungal composition in soils under different fertilization treatments.

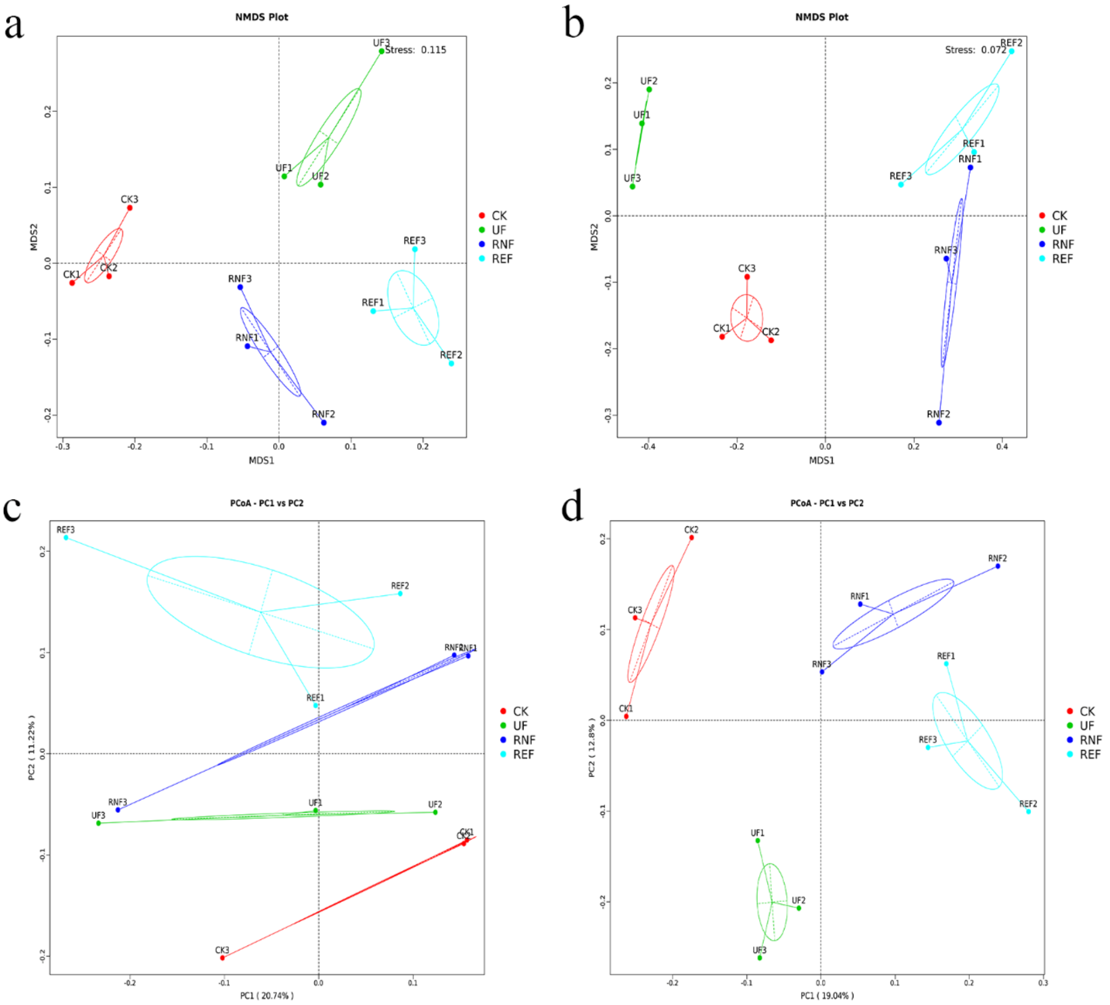

Figure S3 The rose diagrams of relationships between soil key properties and fungal communities in soils with enzymatic fermented rapeseed cake.

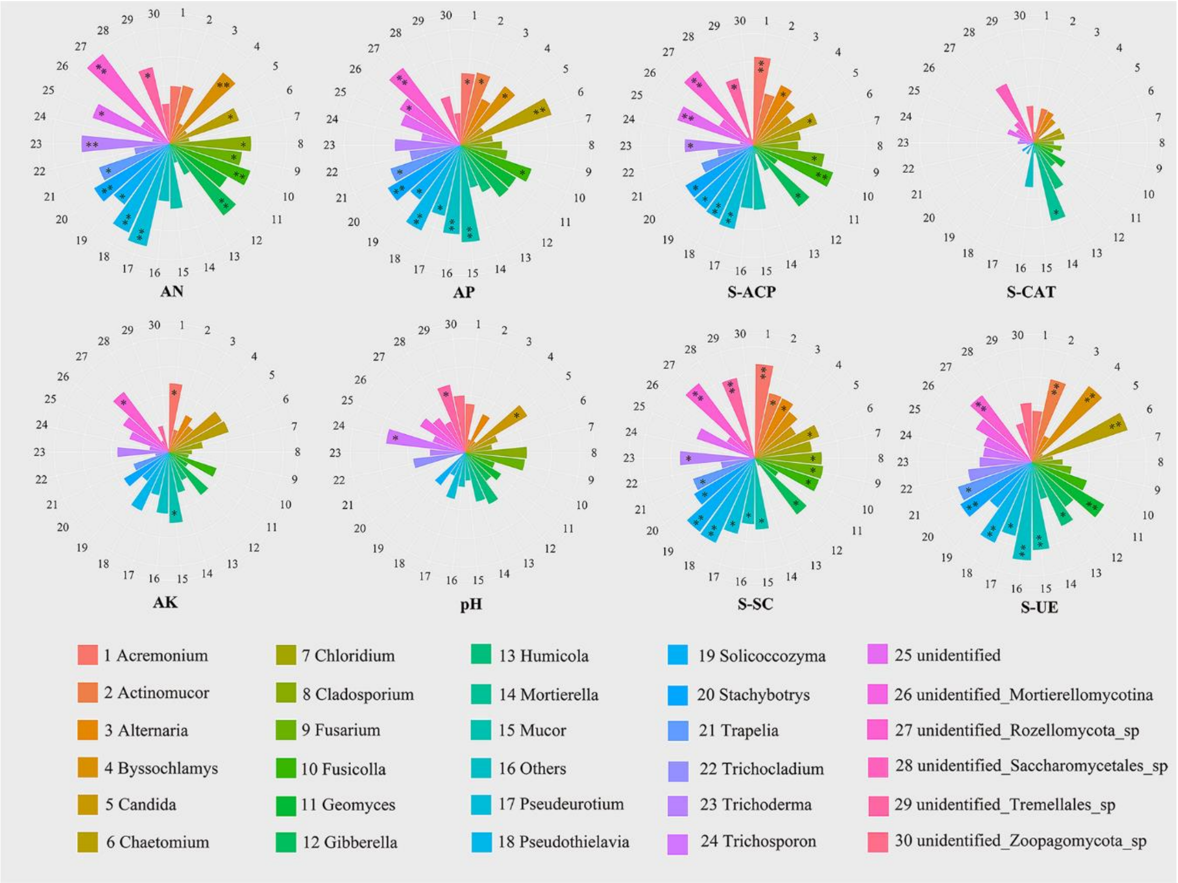

Figure S4 (a) The pie chart of soil metabolite composition. (b)The OPLS-DA analysis of metabolites in CK vs REF. (c)The OPLS-DA analysis of metabolites in UF vs REF. (d)The OPLS-DA analysis of metabolites in RNF vs REF.

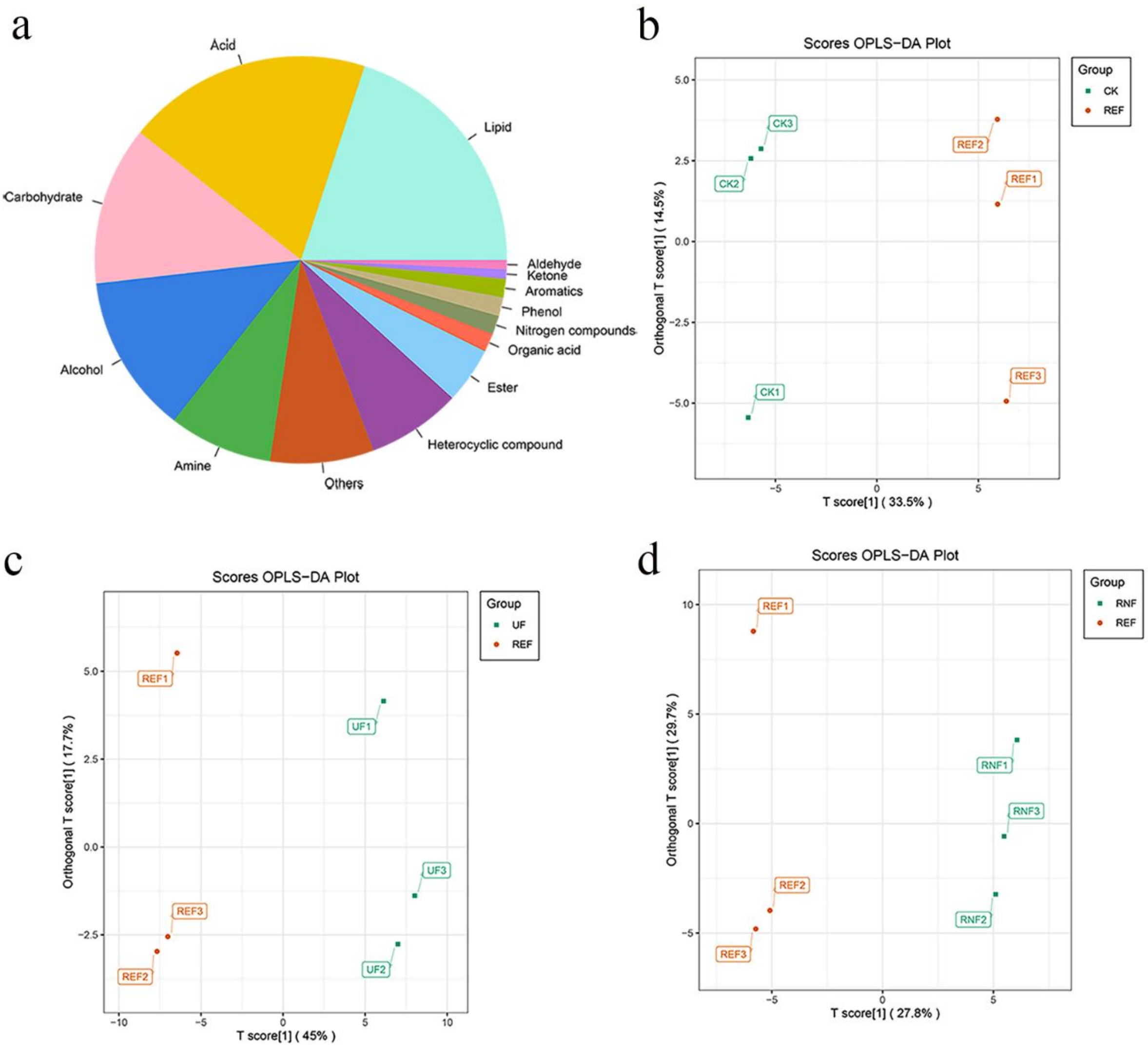

Figure S5 (a) The heatmap of the differential relative content of common metabolites in UF vs REF group. (b) The heatmap of the differential relative content of common metabolites in RNF vs REF group.

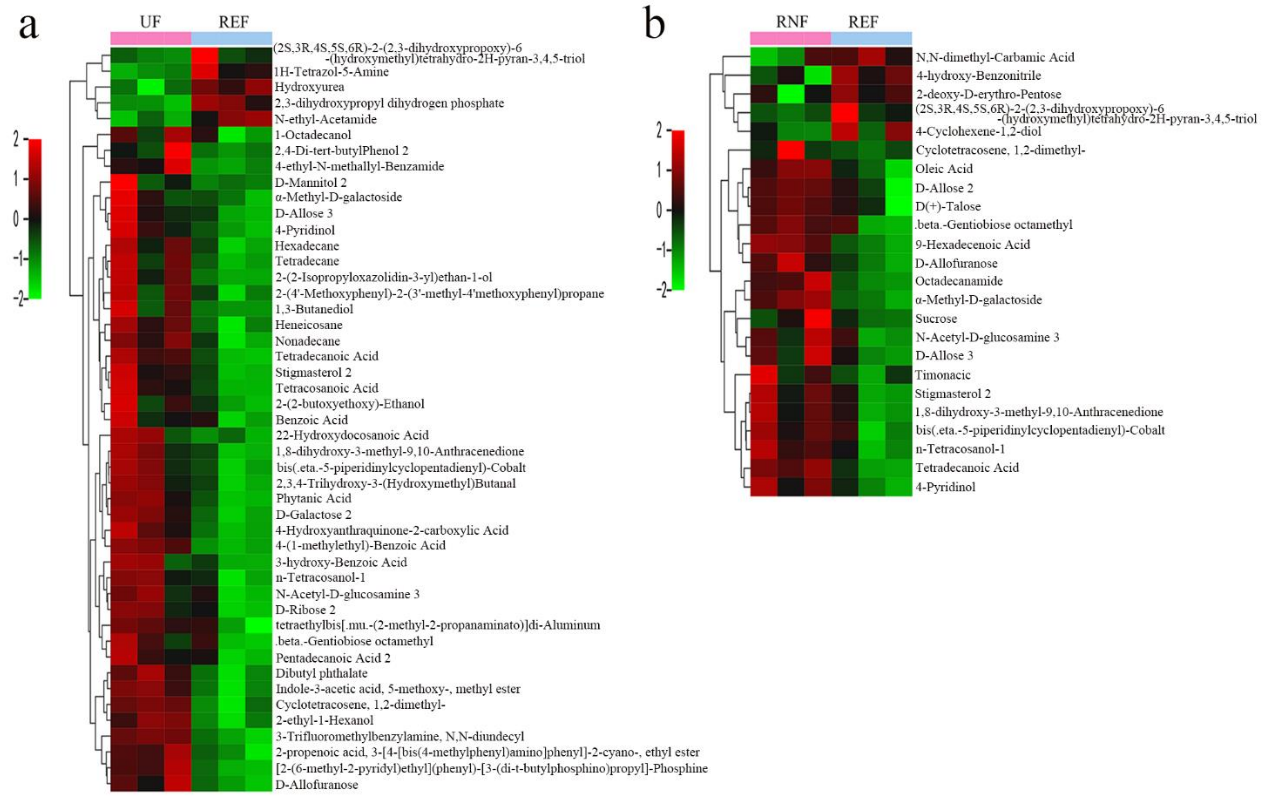

Figure S6 The ellipse heatmap of relationships between fungal communities and soil metabolites.

(a) The relationship of bacterial communities and soil metabolites in CK vs REF group.

(b) The relationship of bacterial communities and soil metabolites in UF vs REF group.

(c) The relationship of bacterial communities and soil metabolites in RNF vs REF group.

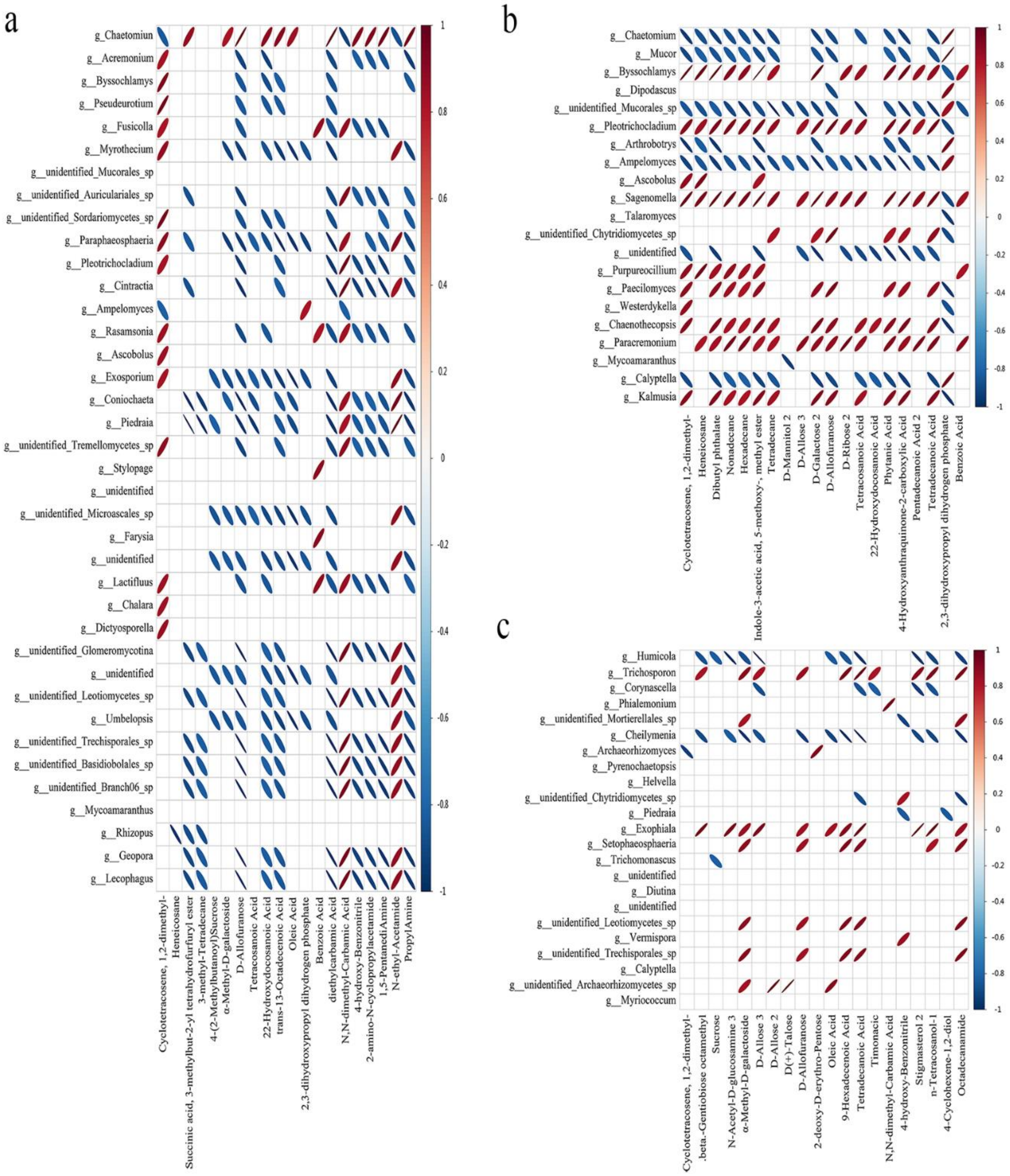

Supplement: Supplementary file 1 — Additional file 1: Figure S1. The Rarefaction curve and Rank abundance of microbial communities in soils under different fertilization treatments. Figure S2. The NMDS and PCoA analysis of microbial communities in soils under different fertilization treatments. Figure S3. The rose diagrams of relationships between soil key properties and fungal communities in soils with enzymatic fermented rapeseed cake. Figure S4. (a) The pie chart of soil metabolite composition. (b) The OPLS-DA analysis of metabolites in CK vs REF. (c) The OPLS-DA analysis of metabolites in UF vs REF. (d) The OPLS-DA analysis of metabolites in RNF vs REF. Figure S5. (a) The heatmap of the differential relative content of common metabolites in UF vs REF. (b) The heatmap of the differential relative content of common metabolites in RNF vs REF. Figure S6. The ellipse heatmap of relationships between fungal communities and soil metabolites. [file 12866_2023_2995_MOESM1_ESM.pdf]
