# Supplementary material for: Enzymatic fermentation of rapeseed cake significantly improved the soil environment of tea rhizosphere
Source: BMC Microbiol. 2023 Sep 7;23:250. doi: 10.1186/s12866-023-02995-7 (PMC10483718; doi:10.1186/s12866-023-02995-7)
Supplement: Supplementary file 2 — Additional file 2: Table S1. Differential metabolite in CK vs REF. Table S2. Differential metabolite in UF vs REF. Table S3. Differential metabolite in RNF vs REF. [file 12866_2023_2995_MOESM2_ESM.pdf]

Table S1 Differential metabolite in CK vs REF

Class	Compounds	VIP	p_value	Fold_Change	Type
Acid	22-Hydroxydocosanoic Acid	1.42	0.07	1.51	up
	N,N-dimethyl-Carbamic Acid	1.63	0.05	0.54	down
	Tetracosanoic Acid	1.36	0.14	1.27	up
	trans-13-Octadecenoic Acid	1.46	0.06	1.94	up
	2,3-dihydroxypropyl dihydrogen phosphate	1.20	0.12	1.27	up
	Oleic Acid	1.35	0.13	1.82	up
	diethylcarbamic Acid	1.68	0.01	1.37	up
	Benzoic Acid	1.06	0.23	0.65	down
Carbohydrate	4-(2-Methylbutanoyl)Sucrose	1.04	0.28	1.46	up
	α-Methyl-D-galactoside	1.31	0.11	3.18	up
	D-Allofuranose	1.70	0.04	15.53	up
Lipid	Succinic acid, 3-methylbut-2-yl tetrahydrofurfuryl ester	1.54	0.05	1.30	up
	3-methyl-Tetradecane	1.42	0.09	1.31	up
	Heneicosane	1.06	0.23	2.21	up
	Cyclotetracosene, 1,2-dimethyl-	1.30	0.11	0.58	down
Alcohol	2,2'-oxybis-Ethanol	1.05	0.20	0.69	down
	Benzyl Alcohol	1.17	0.17	0.63	down
	Myo-Inositol 3	1.22	0.18	2.23	up
	3-chloro-1,2-Propanediol	1.34	0.12	0.57	down
Amine	2-amino-N-cyclopropylacetamide	1.60	0.02	2.10	up
	1,5-PentanediAmine	1.66	0.01	2.93	up
	PropylAmine	1.61	0.01	1.57	up
	N-ethyl-Acetamide	1.49	0.04	0.66	down
Heterocyclic compound	(R)-4-[(5,6,7,8-tetrahydro-1,3-dioxolo[4,5-g]isoquinolin-5-yl)methyl]-Phenol	1.43	0.06	1.39	up
	2-(2-Isopropyloxazolidin-3-yl)ethan-1-ol	1.40	0.06	1.96	up
	(2S,3R,4S,5S,6R)-2-(2,3-dihydroxypropoxy)-6-(hydroxymethyl)tetrahydro-2H-pyran-3,4,5-triol	1.43	0.20	4.78	up
	1-methyl-2-Pyrrolidinone	1.37	0.11	0.45	down
Ester	(1R,2S,5R)-2-isopropyl-5-methylcyclohexyl 2,2 -dihydroxyacetate	1.53	0.03	0.56	down
Nitrogen compounds	Hydroxyurea	1.72	0.01	0.37	down
Aromatics	4-hydroxy-Benzonitrile	1.60	0.01	3.81	up
Others	4-ethyl-N-methallyl-Benzamide	1.52	0.07	0.70	down
	1-(diisopropylphosphino)-3-(diisopropylphosphinyl)-Propane	1.33	0.16	0.28	down

Table S2 Differential metabolite in UF vs REF

Class	Compounds	VIP	p_value	Fold_Change	Type
Acid	Tetracosanoic Acid	1.24	0.053	0.66	down
	22-Hydroxydocosanoic Acid	1.15	0.10	0.52	down
	Phytanic Acid	1.33	0.01	0.60	down
	4-Hydroxyanthraquinone-2-carboxylic Acid	1.36	0.03	0.49	down
	Pentadecanoic Acid 2	1.12	0.09	0.68	down
	Tetradecanoic Acid	1.34	0.01	0.63	down
	2,3-dihydroxypropyl dihydrogen phosphate	1.43	0.01	2.08	up
	4-(1-methylethyl)-Benzoic Acid	1.46	0.01	0.64	down
	3-hydroxy-Benzoic Acid	1.08	0.11	0.61	down
	Benzoic Acid	1.01	0.16	0.57	down
Carbohydrate	.beta.-Gentiobiose octamethyl	1.00	0.16	0.58	down
	N-Acetyl-D-glucosamine 3	1.11	0.10	0.65	down
	α-Methyl-D-galactoside	1.01	0.17	0.42	down
	D-Mannitol 2	1.03	0.24	0.42	down
	D-Allose 3	1.15	0.11	0.50	down
	D-Galactose 2	1.36	0.01	0.57	down
	D-Allofuranose	1.35	0.03	0.38	down
	D-Ribose 2	1.16	0.07	0.60	down
Lipid	2-propenoic acid, 3-[4-[bis(4-methylphenyl)amino]phenyl]-2-cyano-, ethyl ester	1.35	0.014	0.67	down
	Cyclotetracosene, 1,2-dimethyl-	1.36	0.02	0.49	down
	Heneicosane	1.22	0.01	0.36	down
	Dibutyl phthalate	1.36	0.01	0.62	down
	Nonadecane	1.27	0.03	0.48	down
	Hexadecane	1.25	0.04	0.43	down
	Indole-3-acetic acid, 5-methoxy-, methyl ester	1.36	0.02	0.57	down
	Tetradecane	1.27	0.07	0.54	down
Alcohol	Stigmasterol 2	1.25	0.05	0.58	down
	n-Tetracosanol-1	1.21	0.05	0.65	down
	1-Octadecanol	1.07	0.14	0.65	down
	2-(2-butoxyethoxy)-Ethanol	1.10	0.12	0.60	down
	2-ethyl-1-Hexanol	1.38	0.01	0.47	down
	1,3-Butanediol	1.18	0.13	0.61	down
Amine	N-ethyl-Acetamide	1.34	0.01	2.63	up
	1H-Tetrazol-5-Amine	1.25	0.08	1.29	up
Heterocyclic compound	[2-(6-methyl-2-pyridyl)ethyl](phenyl)-[3-(di-t-butylphosphino)propyl]-Phosphine	1.43	0.01	0.67	down
	bis(.eta.-5-piperidinylcyclopentadienyl)-Cobalt	1.25	0.04	0.49	down

	(2S,3R,4S,5S,6R)-2-(2,3-dihydroxypropoxy)-6-(hydroxymethyl)tetrahydro-2H-pyran-3,4,5-triol	1.12	0.24	3.25	up
	2-(2-Isopropyloxazolidin-3-yl)ethan-1-ol	1.37	0.05	0.38	down
	4-Pyridinol	1.23	0.07	0.53	down
Nitrogen compounds	Hydroxyurea	1.31	0.02	1.20	up
Ketone	1,8-dihydroxy-3-methyl-9,10-Anthracenedione	1.24	0.04	0.56	down
Aldehyde	2,3,4-Trihydroxy-3-(Hydroxymethyl)Butanal	1.25	0.03	0.54	down
Phenol	2,4-Di-tert-butylPhenol 2	1.14	0.21	0.69	down
Others	3-Trifluoromethylbenzylamine, N,N-diundecyl	1.43	0.01	0.69	down
	tetraethylbis[.mu.-(2-methyl-2-propanaminato)]di-Aluminum	1.07	0.14	0.64	down
	2-(4'-Methoxyphenyl)-2-(3'-methyl-4'methoxyphenyl)propane	1.09	0.11	0.50	down
	4-ethyl-N-methallyl-Benzamide	1.38	0.08	0.62	down

Table S3 Differential metabolite in RNF vs REF

Class	Compounds	VIP	p_value	Fold_Change	Type
Acid	Oleic Acid	1.49	0.05	0.60	down
	9-Hexadecenoic Acid	1.73	0.01	0.33	down
	Tetradecanoic Acid	1.70	0.02	0.65	down
	Timonacic	1.36	0.15	0.62	down
	N,N-dimethyl-Carbamic Acid	1.32	0.15	1.22	up
Carbohydrate	.beta.-Gentiobiose octamethyl	1.34	0.15	0.58	down
	Sucrose	1.18	0.27	0.36	down
	N-Acetyl-D-glucosamine 3	1.24	0.16	0.68	down
	α-Methyl-D-galactoside	1.69	0.01	0.29	down
	D-Allose 3	1.36	0.13	0.56	down
	D-Allose 2	1.20	0.16	0.59	down
	D(+)-Talose	1.13	0.20	0.61	down
	D-Allofuranose	1.59	0.04	0.47	down
	2-deoxy-D-erythro-Pentose	1.07	0.25	1.22	up
Lipid	Cyclotetracosene, 1,2-dimethyl-	1.26	0.28	0.37	down
Alcohol	Stigmasterol 2	1.46	0.08	0.68	down
	n-Tetracosanol-1	1.20	0.06	0.66	down
	4-Cyclohexene-1,2-diol	1.28	0.18	1.21	up
Amine	Octadecanamide	1.72	0.03	0.69	down
Heterocyclic compound	bis(.eta.-5-piperidinylcyclopentadienyl)-Cobalt	1.32	0.12	0.68	down
	(2S,3R,4S,5S,6R)-2-(2,3-dihydroxypropoxy)-6-(hydroxymethyl)tetrahydro-2H-pyran-3,4,5-triol	1.41	0.25	3.06	up
	4-Pyridinol	1.58	0.04	0.61	down
Ketone	1,8-dihydroxy-3-methyl-9,10-Anthracenedione	1.44	0.08	0.68	down
Aromatics	4-hydroxy-Benzonitrile	1.44	0.10	1.28	up
